# A novel polypeptide encoded by circSPIRE1 promotes prostate cancer proliferation and migration by restraining the ubiquitin-dependent degradation of LRP5

**DOI:** 10.1186/s13046-025-03467-8

**Published:** 2025-07-25

**Authors:** Jintao Hu, Juanyi Shi, Junjie Wang, Yunfei Xiao, Degeng Kong, Mingchao Gao, Tianlong Luo, Shizhong Xu, Zhihan Yuan, Xinyi Ma, Xueseng Dong, Jingang Huang, Cheng Liu, Kewei Xu

**Affiliations:** 1 Department of Urology, Sun Yat-sen Memorial Hospital, Sun Yat-sen University, Guangzhou, 510120 Guangdong China; 2 Guangdong Provincial Key Laboratory of Malignant Tumor Epigenetics and Gene Regulation, Sun Yat-sen Memorial Hospital, Sun Yat-sen University, Guangzhou, 510120 Guangdong China; 3 Guangdong Provincial Clinical Research Center for Urological Diseases, Guangzhou, 510120 Guangdong China; 4 Sun Yat-sen University School of Medicine, Sun Yat-sen University, Shenzhen, 518000 Guangdong China; 5 The Vancouver Prostate Centre, Department of Urologic Sciences, Vancouver General Hospital, The University of British Columbia, 2660 Oak Street, Vancouver, BC V6H3Z6 Canada; 6 Department of Urology, The Fifth Affiliated Hospital of Xinjiang Medical University, Urumqi, 830011 Xinjiang China

**Keywords:** circSPIRE1, Rolling circle translation, Peptide, hnRNPA1, Proliferation, Migration, Prostate cancer, LRP5, Ubiquitination

## Abstract

**Background:**

Circular RNAs (circRNAs) are increasingly implicated in tumor progression, but the roles of protein-coding circRNAs remain largely unexplored. This study characterizes a novel circRNA-encoded protein, rtSPIRE1, and investigates its mechanistic role in prostate cancer proliferation and migration, as well as its diagnostic and therapeutic potential.

**Methods:**

RNA microarray identified circSPIRE1 in prostate cancer tissues. The translation of rtSPIRE1 was confirmed by polysome profiling analysis, Western blotting, and mass spectrometry. Expression levels of circSPIRE1 and rtSPIRE1 were analyzed via qPCR, FISH, and immunohistochemistry. Gain- and loss-of-function assays were performed to evaluate their effects on cell proliferation and migration. Mechanistic studies were conducted using RNA pulldown, RIP, co-immunoprecipitation, and molecular docking.

**Results:**

We identified rtSPIRE1, a novel protein encoded by circSPIRE1 through rolling circle translation, whose expression is regulated by hnRNPA1-mediated symmetric dimethylation. Both circSPIRE1 and rtSPIRE1 were significantly upregulated in prostate cancer tissues and cell lines, with higher expression levels correlating with worse prognosis. Mechanistically, rtSPIRE1 stabilized LRP5 by inhibiting its ubiquitination and degradation, leading to sustained activation of the PI3K/AKT signaling pathway, which ultimately promotes prostate cancer cell proliferation and migration.

**Conclusions:**

Our findings identify rtSPIRE1 as a critical oncogenic protein encoded by circSPIRE1 through rolling circle translation, with its expression regulated by hnRNPA1-mediated symmetric dimethylation. Mechanistically, rtSPIRE1 promotes proliferation and migration by stabilizing LRP5 and activating the PI3K/AKT signaling pathway. Together, circSPIRE1 and rtSPIRE1 represent promising diagnostic biomarkers and therapeutic targets for prostate cancer.

**Supplementary Information:**

The online version contains supplementary material available at 10.1186/s13046-025-03467-8.

## Background

Prostate cancer is the most commonly diagnosed cancer in males worldwide, accounting for 15% of all cancer cases [1]. The global burden of prostate cancer is escalating, with annual incidence projected to surge from 1.4 million cases in 2020 to 2.9 million by 2040 [1, 2]. Among men, it ranks second only to lung cancer as a cause of cancer-related mortality. While the majority of tumors are manageable by radiation therapy or surgical intervention, a significant proportion of tumors progress to incurable locally advanced or metastatic disease [3–6]. Standard treatments, including androgen deprivation therapy (ADT) and modern anti-androgens such as abiraterone and enzalutamide, have improved clinical outcomes but provide only modest survival benefits, particularly in metastatic prostate cancer [7]. Moreover, the significant heterogeneity of prostate tumors, especially in metastatic settings, has limited the effectiveness of emerging therapies like Poly (ADP-ribose) polymerase (PARP) inhibitors and immunotherapies, which benefit only a small subset of patients with advanced or metastatic disease [8–10]. Acordingly, identifying alternative therapeutic targets is essential to developing more effective treatment strategies for metastatic prostate cancer.

Circular RNAs (circRNAs) are a highly conserved class of covalently closed RNAs generated through back-splicing pre-mRNAs, with emerging evidence demonstrating their capacity to encode novel functional peptides [11]. These peptides play critical roles in regulating signaling pathways, promoting epithelial-mesenchymal transition (EMT), and modulating tumor immunity, thereby influencing tumor progression [12–14]. The translation of circRNAs is primarily regulated by internal ribosome entry sites (IRES) or N6-methyladenosine (m6A)-modifications, with IRES being the dominant mechanism. IRES activity is further modulated by IRES-dependent trans-acting factors (ITAFs) [15, 16]. Notably, some coding circRNAs lacking stop codons can undergo rolling circle translation, producing repeated peptide sequences without frameshifting [17]. However, the functional mechanisms of circRNA-encoded peptides in prostate cancer progression remain largely unexplored.

Heterogeneous nuclear ribonucleoprotein A1 (hnRNPA1) is a multifunctional RNA-binding protein within the hnRNP family, involved in RNA metabolism, including splicing, transport, stability, and translation [18, 19]. It regulates RNA translation by binding to IRES, particularly under stress conditions. hnRNPA1 contains exposed arginine (R) residues that undergo post-translational modifications crucial for IRES activity regulation [20–22]. However, the mechanisms by which hnRNPA1, through specific post-translational modifications, mediates circRNA IRES activity and drives prostate cancer progression remain unknown.

In this study, we identified a novel coding circRNA, circSPIRE1, derived from the SPIRE1 gene through back-splicing and highly expressed in prostate cancer. circSPIRE1 undergoes rolling circle translation via an IRES-dependent mechanism, which is regulated by hnRNPA1. Notably, post-translational modifications of hnRNPA1 differentially modulate the IRES activity of circSPIRE1. This translation process generates a set of proteins termed rtSPIRE1, characterized by repeated peptide sequences encoded by the open reading frame (ORF), with primary products comprising up to three repeats. We further demonstrated that rtSPIRE1 interacts with LDL receptor-related protein 5 (LRP5), inhibiting its ubiquitination and thereby activating the PI3K/AKT pathway. This signaling cascade ultimately drives prostate cancer progression. Our findings reveal a novel mechanism by which circSPIRE1, regulated by post-translational modification of hnRNPA1, encodes rtSPIRE1 to activate PI3K/AKT signaling, thereby promoting prostate cancer progression. These results identify circSPIRE1 as a potential therapeutic target, providing a potential avenue for improved prostate cancer management.

## Methods

### Patients and clinical samples

Prostate cancer tumor specimens, along with matched adjacent non-tumor tissues, were collected from patients undergoing radical prostatectomy at the Department of Urology, Sun Yat-sen Memorial Hospital, Sun Yat-sen University (Guangzhou, China), between January 2010 and January 2020. Pathological examination confirmed all samples, which were preserved in RNAlater and stored at − 80 °C immediately after collection. This study received approval from the Sun Yat-sen Memorial Hospital Ethical Review Committee, and all participating patients provided written informed consent.

### Cell lines, cell culture, and reagents

Human prostate cancer cell lines (DU145, PC-3) and HEK-293T cells were obtained from the American Type Culture Collection (ATCC, Manassas, VA, USA). Cells were cultured in the recommended medium supplemented with 10% Gibco fetal bovine serum (Thermo Fisher Scientific) under standard conditions (37℃, 5% CO2, humidified atmosphere). Cell lines were authenticated by short tandem repeat analysis within two years of the study. The total number of passages for these cell lines was kept below 15 from thawing to use in this study. Mycoplasma contamination was routinely tested after cell revival using the Mycoalert Mycoplasma Detection Kit (Beyotime).

For experimental treatments, MG-132 (100 nM; Selleck Chemicals, S2619), cycloheximide (CHX, 50 µg/mL; Selleck Chemicals, S7418), and EPZ015666 (10 µM; MedChemExpress, HY-12727) were used. Human recombinant hnRNPA1 protein (MedChemExpress, HY-P72229) was utilized in binding assays following the manufacturer’s protocol.

### RNA interference and transfection

Small interfering RNAs (siRNAs) targeting circSPIRE1, hnRNPA1, and LRP5, along with a control siRNA, were designed and synthesized by GenePharma (Shanghai, China). Transfections were performed using Lipofectamine™ RNAiMAX Transfection Reagent (Thermo Fisher Scientific, 13778150) and Opti-MEM^®^ I Reduced Serum Medium (Thermo Fisher Scientific, 31985-062) according to the manufacturer’s protocol. Cells were plated in a 6-well plate one day prior to transfection, ensuring that cell confluence reached 50–70% the following day. RNAi duplexes were diluted to 30 pmol in 50 µl Opti-MEM and mixed gently. Lipofectamine™ RNAiMAX was diluted by adding 1.5 µl to 50 µl Opti-MEM, and the complexes were incubated for 10–20 min at room temperature. The RNAi duplex-Lipofectamine™ RNAiMAX complexes were then added to the cells, resulting in a final siRNA concentration of 50 nM, which is typically used, though the concentration may be optimized depending on experimental conditions. Cells were incubated under standard culture conditions for 24–48 h before gene knockdown assays. The specific siRNA sequences are listed in Table S6.

### Plasmid and ShRNA constructs and transfection

The circSPIRE1-Flag, circSPIRE1-Flag-mut, and Vector plasmids were synthesized by Geneseed Biotech (Guangzhou, China). Additional constructs, including circSPIRE1 overexpression (circSPIRE1-OE), shRNAs targeting circSPIRE1, and control sequences, were obtained from IGE BIO (Guangzhou, China). For translational studies, plasmids containing varied open reading frames (1R, 2R, 3R, and 4R) and hnRNPA1-HA plasmids, along with site-directed hnRNPA1-HA mutants, were utilized. All plasmids were amplified in *E. coli* and purified using the EndoFree Plasmid Midi Kit (CWBIO, CW2105S). Transfections were performed using Lipofectamine 3000 (Invitrogen) in Opti-MEM™ medium (Thermo Fisher Scientific, L3000015).

### RNA extraction and RT-qPCR analysis

Total RNA was extracted using TRIzol™ Reagent (Thermo Fisher Scientific, 15596-018) according to the manufacturer’s protocol. For reverse transcription (RT), we used the Hifair^®^ III 1st Strand cDNA Synthesis SuperMix for qPCR (gDNA digester plus) (Yeasen, 11141ES60). For qPCR, we performed the analysis using the Hieff^®^ qPCR SYBR^®^ Green Master Mix (Yeasen, 11185ES08). Both the reverse transcription and qPCR reactions were conducted following the standard procedures recommended by manufacturers. The specific primer sequences used for analysis are provided in Table S6.

### RNA microarray and transcriptome sequencing

RNA microarray analysis was performed by Aksomics (Shanghai, China). Raw data were processed to remove low-quality probes and background noise. The data were then normalized using log2 transformation and quantile normalization to ensure comparability across samples. Differentially expressed circRNAs were identified based on a fold change ≥ 2 and a P-value < 0.01 in comparisons between samples or groups. Transcriptome sequencing was conducted by IGE BIO (Guangzhou, China). The data were processed using standard quality control steps, and the normalization procedure included RPKM/FPKM for RNA-seq data to ensure accurate comparison between samples.

### Actinomycin D, RNase R, and nuclear-cytoplasmic fractionation

Cells were seeded in 24-well plates and treated with 2 µg/mL actinomycin D (Abmole) after 24 h of cultivation for 0, 8, 16, and 24 h. Total RNA was extracted at each time point, and RT-qPCR was performed to evaluate the stability of circRNA and mRNA expression. For RNase R treatment, total RNA was incubated at 37 ℃with or without RNase R (Beyotime Biotechnology) for 30 min, followed by RT-qPCR to verify circRNA stability. GAPDH served as the internal control in RNase R-free samples. Nuclear and cytoplasmic fractionation was performed using the PARIS™ Kit (Invitrogen™) according to the manufacturer’s protocol.

### Polysome purification analysis

Cells were plated in 10 cm dishes and cultured to reach 40–50% confluency after 24 h. Transfection was performed using 10 µg of plasmid and Lipofectamine 3000. After 48 h, cells were treated with 100 µg/mL cycloheximide in DMSO and harvested for polysome analysis. Approximately 5–6 × 10^7 cells were lysed in polysome lysis buffer containing 100 µg/mL cycloheximide and EDTA-free protease inhibitors on ice for 15 min. The lysate was centrifuged at 12,000×g for 10 min at 4 ℃ to remove nuclei and mitochondria. The cleared supernatant was layered onto a 10–50% sucrose gradient and subjected to ultracentrifugation at 20,000×g for 2 h at 4 ℃. Fractions were collected, and RNA was extracted from each fraction using TriZol reagent.

### Dual-Luciferase assay system

To evaluate the internal ribosome entry site (IRES) activity of circSPIRE1, 2 × 10⁵ cells were seeded in 6-well plates and transfected with 2 µg of dual-luciferase reporter construct plasmid, which was obtained from Geneseed Biotech (Guangzhou, China). Transfection was performed using Lipofectamine™ 3000 reagent (Thermo Fisher Scientific, L3000015) according to the manufacturer’s instructions. After 48 h of incubation, luciferase activities were measured using the Dual-Luciferase Reporter Assay System (Yeasen, 11402ES60). Firefly luciferase activity was measured first, followed by Renilla luciferase activity. The results were normalized by calculating the ratio of firefly to Renilla luciferase activity. The calculation was performed as follows:

$$\begin{gathered}{\text{Experimental}}{\mkern 1mu} {\text{Group}}{\mkern 1mu} {\text{Ratio}}{\mkern 1mu} \hfill \\= {\mkern 1mu} \frac{{\left( {\begin{aligned}&{\text{Experimental}}{\mkern 1mu} {\text{Group}}{\mkern 1mu} {\text{Firefly}}{\mkern 1mu} \cr&\quad- {\mkern 1mu} {\text{Background}}{\mkern 1mu} {\text{Firefly}}\end{aligned}} \right)}}{{\left( {\begin{aligned}&{\text{Experimental}}{\mkern 1mu} {\text{Group}}{\mkern 1mu} {\text{Renilla}}{\mkern 1mu} \cr&\quad- {\mkern 1mu} {\text{Background}}{\mkern 1mu} {\text{Renilla}}\end{aligned}} \right)}}{\mkern 1mu} \hfill \\\end{gathered} $$

$$\begin{gathered}{\text{Control}}{\mkern 1mu} {\text{Group}}{\mkern 1mu} {\text{Ratio}}{\mkern 1mu} \hfill \\= {\mkern 1mu} \frac{{\left( {{\text{Control}}{\mkern 1mu} {\text{Group}}{\mkern 1mu} {\text{Firefly}}{\mkern 1mu} - {\mkern 1mu} {\text{Background}}{\mkern 1mu} {\text{Firefly}}} \right)}}{{\left( {{\text{Control}}{\mkern 1mu} {\text{Group}}{\mkern 1mu} {\text{Renilla}}{\mkern 1mu} - {\mkern 1mu} {\text{Background}}{\mkern 1mu} {\text{Renilla}}} \right)}} \hfill \\\end{gathered} $$

$$\begin{gathered}{\text{Expression}}{\mkern 1mu} {\text{Fold}}{\mkern 1mu} {\text{Change}}{\mkern 1mu} \hfill \\= {\mkern 1mu} \frac{{{\text{Experimental}}{\mkern 1mu} {\text{Group}}{\mkern 1mu} {\text{Ratio}}}}{{{\text{Control}}{\mkern 1mu} {\text{Group}}{\mkern 1mu} {\text{Ratio}}}} \hfill \\\end{gathered} $$

Detection was carried out using a luminescence detection system with a wavelength range of 350–700 nm. The detection time was set between 2 and 10 s.

### FISH and Immunofluorescence

Cy3-labeled probes targeting the circSPIRE1 junction were synthesized by GenePharma (Shanghai, China), with sequences provided in Table S6. For immunofluorescence (IF) staining, the rtSPIRE1 antibody was custom-prepared by HUABIO (China), while FLAG (Sigma, F1804) and LRP5 (CST, 5731) antibodies were used. The Cy3-labeled probes were detected with an excitation wavelength of 550 nm and emission at 570 nm. DAPI (Servicebio, G1012) was used for nuclear staining, with excitation at 350–400 nm and emission at 450–480 nm. For IF staining, the mouse-derived anti-FLAG antibody was detected using a DyLight 594-conjugated secondary antibody (Abbkine, A23420), and the rabbit-derived anti-LRP5 antibody was detected using a DyLight 488-conjugated secondary antibody (Abbkine, A23230). Images were acquired using an Olympus FV3000 confocal microscope, and analysis was performed using ImageJ software (National Institutes of Health, USA).

### Western blotting and proteome profiler assay

Whole-cell lysates were separated by SDS-PAGE and transferred onto PVDF membranes (Millipore). Membranes were incubated with the following primary antibodies: anti-FLAG (1:1000, Sigma, F1804), anti-GAPDH (1:50000, Proteintech, 60004-1-lg), anti-hnRNPA1 (1:5000, Proteintech, 11176-1-AP), anti-SDMA (1:1000, CST, 13222), anti-HA (1:5000, Proteintech, 51064-2-AP), custom anti-rtSPIRE1 (1:500, rabbit-derived, HUABIO), anti-pan-AKT (1:1000, CST, 4691), anti-p-AKT (Thr308, 1:1000, CST, 13038; Ser473, 1:1000, CST, 4060), anti-mTOR (1:1000, CST, 2972), anti-p-mTOR (Ser2448, 1:1000, CST, 2971), anti-E-cadherin (1:2000, Proteintech, 60335), anti-N-cadherin (1:5000, Proteintech, 66219), anti-vimentin (1:20000, Proteintech, 80232), anti-LRP5 (1:1000, CST, 5731), and anti-ubiquitin (1:200, Santa Cruz, sc8017). HRP-conjugated secondary antibodies (1:2000, anti-mouse IgG, Proteintech, SA00001-1; 1:2000, anti-rabbit IgG, Proteintech, SA00001-2) were used, and signals were detected using an ECL system (Millipore).

Phosphorylation levels were assessed using the Human Phospho-Kinase Array Kit (ARY003B, R&D Systems) following the manufacturer’s instructions.

### RNA pulldown, immunoprecipitation, and RNA Immunoprecipitation

RNA pulldown assays were performed using the Pierce™ Magnetic RNA-Protein Pull-Down Kit (Thermo Fisher Scientific) according to the manufacturer’s protocol. Probes targeting the specific splice junction of circSPIRE1 were designed and synthesized by GenePharma (Guangzhou, China), with sequences provided in Table S6, to ensure selective binding.

Immunoprecipitation (IP) experiments were conducted using the Thermo Fisher IP Kit. For RIP assays, the Magna RIP™ RNA-Binding Protein Immunoprecipitation Kit (MilliporeSigma) was employed following the manufacturer’s instructions.

### Molecular Docking analysis

Molecular docking was performed to investigate the interactions of circSPIRE1 with hnRNPA1 and rtSPIRE1 with LRP5.

For the hnRNPA1 interaction study, the hnRNPA1 protein model (UniProt ID: P09651) was retrieved from UniProt. Docking was conducted using the ZDOCK SERVER, with docking scores above 1000 indicating binding potential. Scores of 1250 were considered strong, while scores over 1400 were regarded as optimal. The top 10 poses were generated, and the highest-scoring model was selected as the optimal interaction structure. Protein preparation, including the removal of water molecules and addition of hydrogen atoms, was performed using PyMol 2.4.

For the rtSPIRE1-LRP5 interaction study, the LRP5 protein model (UniProt ID: O75197) was obtained from UniProt, and the rtSPIRE1 peptide model was generated using AlphaFold2, selecting the model with the highest pLDDT score. Docking was carried out using the HDOCK SERVER, with binding scores of 200 indicating moderate binding, 250 representing strong binding, and 300 or above suggesting optimal binding. Binding interactions were prepared and visualized using PyMol 2.4.

### Cell viability, Edu, wound healing, and transwell assays

Cell viability was assessed using the CCK-8 assay and colony formation assay. For the CCK-8 assay, 1 × 10³ cells were seeded per well in 96-well plates, and viability was measured at 24 h, 48 h, and 72 h using the CCK-8 kit (APExBIO, K1018) according to the manufacturer’s protocol. Colony formation was assessed by seeding 1 × 10³ cells per well in 6-well plates and culturing for 10–14 days. Colonies were then fixed with 4% paraformaldehyde, stained with 0.1% crystal violet, and colonies containing more than 50 cells were counted manually or using ImageJ. Cell proliferation was evaluated using the BeyoClick™ EdU assay kit (Beyotime, C0075S or C0071S). Cells (2 × 10⁴ to 5 × 10⁴ per well) were seeded in 24-well plates, and incubated with 10 µM EdU for 2 h during the logarithmic growth phase, followed by fixation, permeabilization, and staining per the manufacturer’s instructions. Cell migration and invasion capacities were assessed using wound healing and Transwell assays. For wound healing, cells were seeded in 6-well plates (1 × 10⁵–2 × 10⁵ cells/well) and cultured to near confluence. A scratch was made using a sterile 200 µL pipette tip, and images were taken at 0 and 24 h. For Transwell assays, 2 × 10⁵ cells in serum-free medium were placed into the upper chambers of Falcon 8.0 μm pore-size inserts (#353097). In invasion assays, the chambers were pre-coated with Matrigel (Corning, #354262). After 24 h, migrated or invaded cells on the lower surface were fixed, stained with 0.1% crystal violet, and counted in five random microscopic fields. Quantitative data were analyzed using ImageJ or GraphPad Prism, based on three independent biological replicates.

### Mass spectrum analysis

Protein samples were prepared following Coomassie blue and silver staining and subsequently sent to Junhui Biotechnology (Guangzhou, China) for further analysis.

### Animal experiments

All animal experiments were approved by the Animal Ethics Committee of Sun Yat-sen University and conducted at the university’s Animal Experiment Centre (Guangzhou, China). The study adhered to all relevant guidelines and regulations outlined in the approved protocol. Male BALB/c nude mice (4–6 weeks old) were obtained from the Animal Experiment Centre of Sun Yat-sen University and housed under specific pathogen-free (SPF) conditions with a controlled temperature (22 ± 2 °C), humidity (50–60%), and a 12-hour light/dark cycle. Mice had free access to autoclaved food and water.

For the subcutaneous xenograft model, 2 × 10⁶ tumor cells in 100 µL PBS were injected subcutaneously into the right flank of each mouse. For the bone metastasis model, 1 × 10⁶ tumor cells in 50 µL PBS were administered via tail vein injection. Tumor growth was monitored every 3 days using a digital caliper, and tumor volume was calculated using the standard formula: volume = (length × width²)/2. Each group consisted of 5–8 mice, with equal numbers assigned to experimental and control groups. Data were collected and analyzed statistically. The maximum tumor diameter allowed by the ethics committee (20 mm) was not exceeded during the course of the experiment. Tumor growth data, including figures showing tumor size, will be provided in compliance with the journal’s data availability policy.

### Statistical analysis

Statistical analyses were performed using GraphPad Prism 10.0 (GraphPad Software, Inc., San Diego, CA, USA). Data are expressed as mean ± SD from three independent experiments. For normally distributed data, two-tailed Student’s t-tests or one-way/two-way ANOVA were applied. For non-normally distributed data, the Mann–Whitney U test was used. Survival analyses were conducted using the Kaplan–Meier method, and significance was evaluated with the log-rank test in R (version 4.4.0, www.r-project.org). A p-value < 0.05 was considered statistically significant.

## Results

### circSPIRE1 recognition and characterization in prostate cancer

**Fig. 1:**
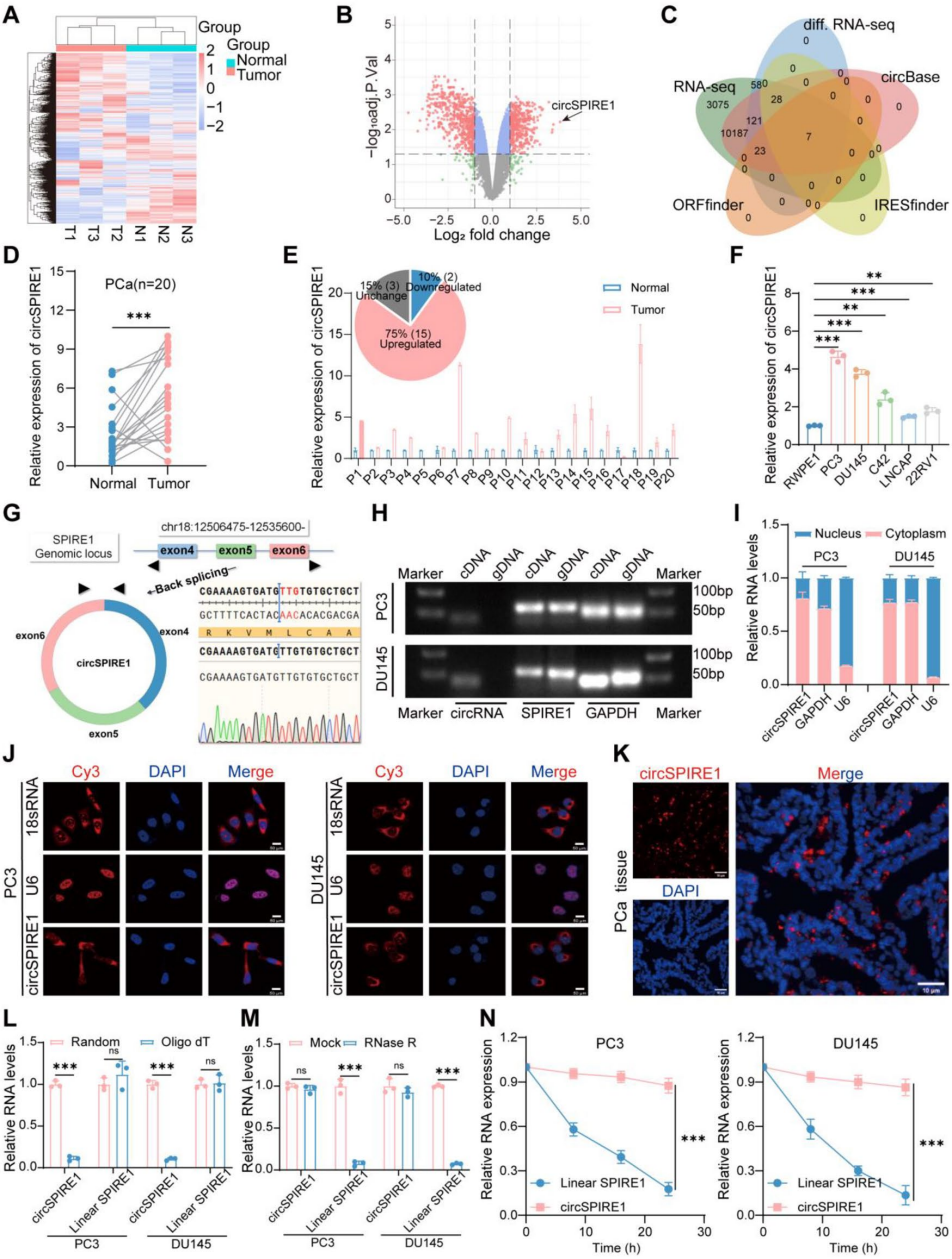
Identification and characteristics of circSPIRE1. (**A**-**B**) Heatmap and volcano plot of circRNA microarray analysis showing differential expression in paired prostate cancer and adjacent tissues. (**C**) Venn diagram illustrating the screening of differentially expressed circRNAs with translational potential, identifying seven circRNAs. (**D**-**E**) RT-qPCR analysis showing upregulation of circSPIRE1 in prostate cancer tissues (*n* = 20). (**F**) RT-qPCR validation of circSPIRE1 expression in prostate cancer cell lines, with highest expression in PC3 and DU145 (*n* = 3). (**G**) Divergent primers amplified circSPIRE1 back-splice junction, confirmed by Sanger sequencing. (**H**) Gel electrophoresis showing circSPIRE1 amplification in cDNA but not in genomic DNA (gDNA) (*n* = 3). (**I**) Nuclear-cytoplasmic fractionation and RT-qPCR show circSPIRE1 is predominantly cytoplasmic (*n* = 3). (**J**) FISH analysis of circSPIRE1 confirming cytoplasmic localization in prostate cancer cell lines (PC3 and DU145). Scale bar, 50 μm (*n* = 3). (**K**) FISH analysis confirming cytoplasmic localization in prostate cancer tissue sections (*n* = 3). Scale bar, 10 μm (*n* = 3). (**L**) RT-qPCR with random and oligo(dT) primers shows circSPIRE1 is non-polyadenylated (*n* = 3). (**M**) RNase R digestion confirms circSPIRE1’s circular form (*n* = 3). (**N**) Actinomycin D assay shows circSPIRE1 has a longer half-life than linear SPIRE1 (*n* = 3). ns: no significance, *p < 0.05, **p < 0.01, ***p < 0.001

To explore the potential role of circRNAs in prostate cancer progression, high-throughput circRNA microarray analysis was performed on cancerous and adjacent normal tissues from three prostate cancer patients (Fig. 1A). A total of 45 circRNAs were significantly upregulated (fold change > 2, *p* < 0.05), while 192 circRNAs were downregulated in prostate cancer samples (Fig. 1B and Table S1-S2). Further screening of circBase-annotated differentially expressed circRNAs using ORFfinder and IRESfinder identified seven circRNAs with translational potential, including hsa_circ_0000829 (circSPIRE1), hsa_circ_0003099, hsa_circ_0016476, hsa_circ_0025402, hsa_circ_0038011, hsa_circ_0005039, and hsa_circ_0082326 (Fig. 1C). Real-time quantitative PCR (RT-qPCR) validation in 20 paired prostate cancer and adjacent normal tissues confirmed the differential expression of these circRNAs, with circSPIRE1 showing the most significant upregulation (Fig. 1D-E, Fig. S1A and Table S3). Additional RT-qPCR in the normal prostate cell line RWPE1 and prostate cancer cell lines PC3, DU145, C42, LNCaP, and 22RV1 revealed consistently elevated circSPIRE1 expression in cancer cell lines, with the highest levels observed in PC3 and DU145 (Fig. 1F).

circSPIRE1, a 369-nucleotide circRNA, is generated through back-splicing of exons 4–6 from SPIRE1 pre-mRNA. The back-splice junction was confirmed by Sanger sequencing using divergent primers (Fig. 1G). RT-qPCR amplification followed by agarose gel electrophoresis showed that circSPIRE1 was detectable only in complementary DNA (cDNA) samples, with no amplification in genomic DNA (gDNA) samples from both PC3 and DU145 cell lines (Fig. 1H). Nuclear-cytoplasmic fractionation and fluorescence in situ hybridization (FISH) indicated that circSPIRE1 is predominantly localized in the cytoplasm in both prostate cancer cell lines and tissues (Fig. 1I-K). RT-qPCR analysis with random and oligo(dT) primers confirmed the non-polyadenylated structure of circSPIRE1, as it was not efficiently amplified by oligo(dT) primers (Fig. 1L). RNase R digestion and actinomycin D treatment demonstrated the enhanced stability of circSPIRE1 compared to linear SPIRE1 RNA (Fig. 1M-N). These results indicated that circSPIRE1, localized in the cytoplasm, is a stable circular RNA formed through back-splicing.

### circSPIRE1 expression is correlated with clinicopathological features in prostate cancer

**Fig. 2:**
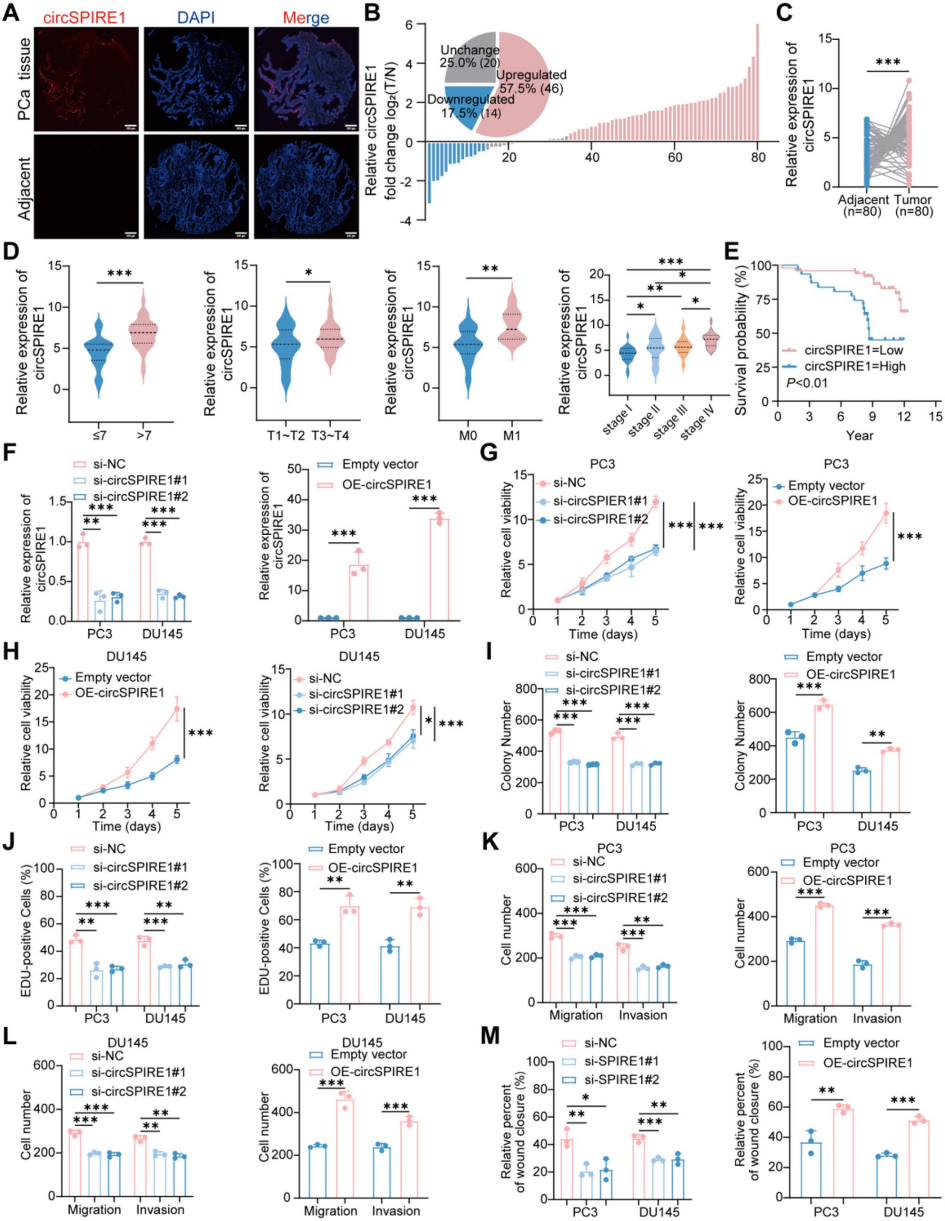
circSPIRE1 correlates with clinical features and promotes prostate cancer progression. (**A**) FISH images showing circSPIRE1 expression (red) in prostate cancer (PCa) tissues and adjacent non-cancerous tissues. Nuclei are stained with DAPI (blue). Scale bar: 200 μm. (**B**) Log2 fold change of circSPIRE1 expression (tumor/normal) in 80 prostate cancer patients: 57.50% upregulated, 17.50% downregulated, and 25.00% unchanged. (**C**-**E**) Quantitative analysis of circSPIRE1 expression by FISH in prostate cancer tissues versus adjacent tissues (**C**), and its correlation with Gleason score (≤ 7 vs. >7), tumor stage (T1–T2 vs. T3–T4), metastasis status (M0 vs. M1), and clinical stage (I–IV) (*n* = 80). (**F**) RT-qPCR validation of circSPIRE1 knockdown and overexpression in prostate cancer cell models (*n* = 3). (**G**-**H**) CCK8 assay assessing cell viability and proliferation in PC3 and DU145 cells (*n* = 3). (**I**) Colony formation assay evaluating colony-forming ability (*n* = 3; see Fig. S1B for representative images). (**J**) EdU assay measuring cell proliferation (*n* = 3; see Fig. S1C for representative images). (**K**-**L**) Transwell assays assessing cell migration and invasion (*n* = 3; see Fig. S2A for representative images). (M) Wound healing assay assessing cell migration (*n* = 3; see Fig. S2B). ns, no significance; *p < 0.05, **p < 0.01, ***p < 0.001

To further investigate the expression pattern and clinical significance of circSPIRE1 in prostate cancer, FISH analysis revealed that circSPIRE1 was markedly overexpressed in prostate cancer tissues (Fig. 2A) and upregulated in the majority of cancer tissues across 80 patient samples (Fig. 2B and C). Elevated circSPIRE1 expression significantly correlated with higher Gleason scores (> 7), advanced T stages, M1 status, and Stage I-IV progression (Fig. 2D). Detailed associations between circSPIRE1 expression levels and clinicopathological parameters are summarized in Table S4. Moreover, patients with high circSPIRE1 expression exhibited poorer prognosis (Fig. 2E).

### circSPIRE1 promotes tumor proliferation and metastasis in prostate cancer

To preliminarily explore the biological function of circSPIRE1, prostate cancer cell models were constructed with gain- and loss-of-function of circSPIRE1 (Fig. 2F). CCK8 assays demonstrated that circSPIRE1 knockdown significantly reduced cell viability, whereas its overexpression markedly enhanced proliferation (Fig. 2G-H). Consistent findings were observed in colony formation and EdU assays: knockdown of circSPIRE1 significantly reduced both colony numbers and EdU-positive cells, whereas overexpression markedly increased them (Fig. 2I-J and Fig. S1B-C). These experiments indicated that circSPIRE1 promotes prostate cancer cell proliferation.

Transwell and wound healing assays indicated that circSPIRE1 knockdown significantly suppressed cell migration and invasion, while overexpression promoted these abilities (Fig. 2K-M and Fig. S2A-B). In vivo, xenograft experiments in nude mice revealed that tumors derived from circSPIRE1-overexpressing cells grew significantly larger in volume and weight compared to controls (Fig. S2C-E). Immunohistochemical staining of these tumors confirmed higher expression of the proliferation marker Ki-67 and mesenchymal markers N-cadherin and vimentin, alongside reduced levels of E-cadherin, suggesting increased tumor aggressiveness and enhanced EMT characteristics (Fig. S2F).

### circSPIRE1 encodes the repetitive peptide rtSPIRE1 through rolling circle translation

**Fig. 3:**
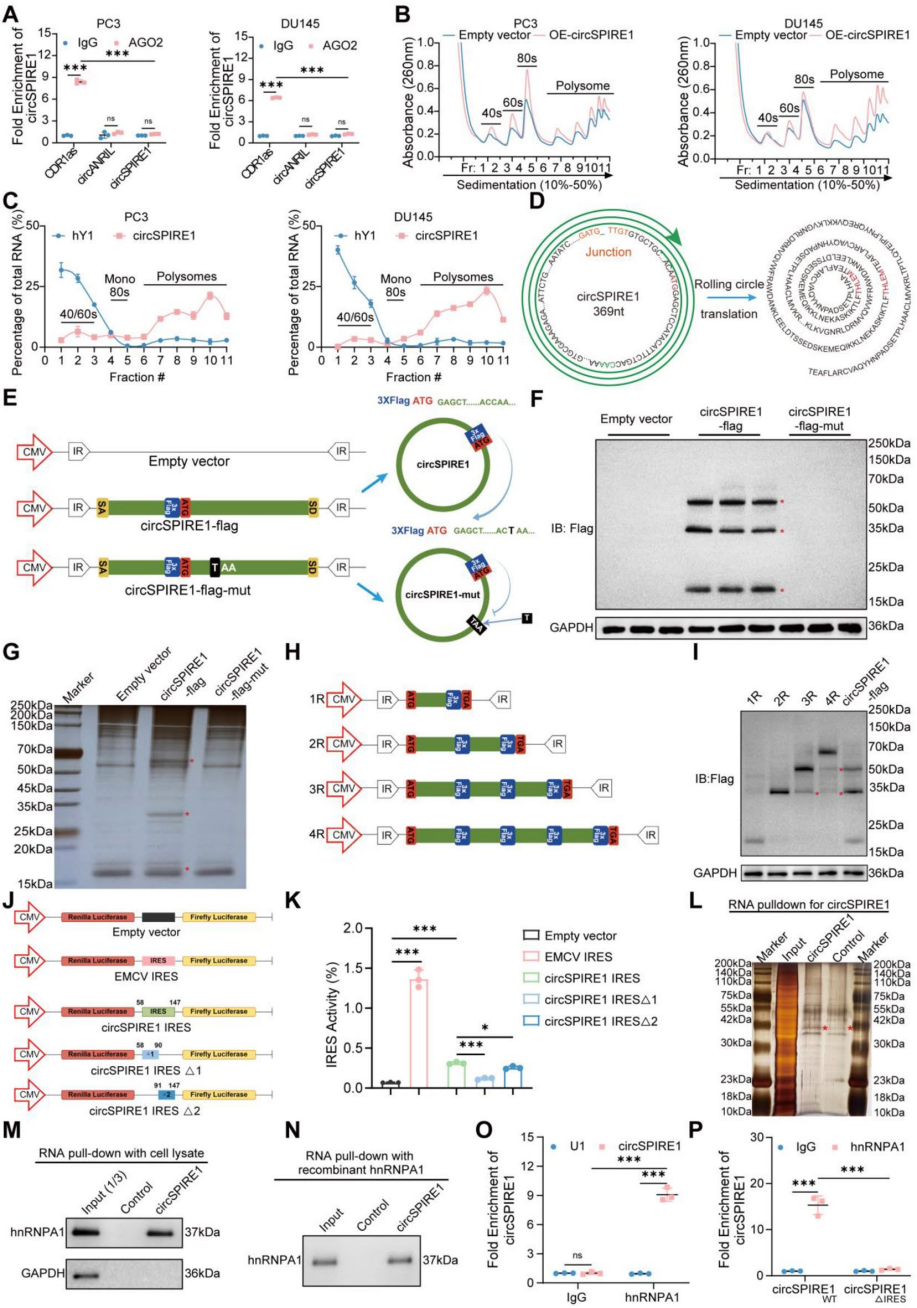
circSPIRE1 drives biological functions through IRES-mediated rolling circle translation. (**A**) RIP assay showing weak AGO2 binding to circSPIRE1 in PC3 and DU145 cells, with CDR1as as a positive control and circANRIL as a negative control (*n* = 3). (**B**) Sucrose gradient centrifugation reveals enhanced association of circSPIRE1 with polysomal fractions upon overexpression in PC3 and DU145 cells. (**C**) Ribosomal subunit distribution of circSPIRE1, showing predominant association with polysomes in PC3 and DU145, with hY1 as a negative control (*n* = 3). (**D**) TransCirc database analysis identifies an open reading frame (ORF) in circSPIRE1, initiated by an ATG codon but lacking a termination codon. (**E**) Constructs designed to evaluate translational potential of circSPIRE1, including circSPIRE1-Flag and circSPIRE1-Flag-mut. (**F**) Western blot analysis reveals three predominant protein bands (17 kDa), consistent with ORF loop translations (*n* = 3). (**G**) SDS-PAGE and mass spectrometry confirm the protein bands correspond to peptides encoded by circSPIRE1, designated as rtSPIRE1. (**H**) Schematic of linear constructs designed with repeated ORFs. (**I**) Western blot analysis of linear constructs 1R, 2R, 3R, and 4R (*n* = 3). (**J**) Schematic of dual-luciferase constructs to evaluate circSPIRE1 IRES activity. (**K**) Relative Luc/Rluc activity shows robust IRES activity in the full-length circSPIRE1 IRES, significantly higher than in other constructs. (**L**) RNA pulldown with a circSPIRE1-specific probe identifies hnRNPA1 as a binding protein. (**M**-**N**) Western blot validation of RNA pulldown assays confirms selective binding of hnRNPA1 to circSPIRE1 (*n* = 3). (**O**-**P**) RIP assays show enhanced binding of hnRNPA1 to wild-type circSPIRE1 and decreased binding to the IRES-deleted mutant (*n* = 3). ns, no significance; *p < 0.05, **p < 0.01, ***p < 0.001

To investigate whether circSPIRE1 primarily functions through protein translation rather than acting as a miRNA sponge, RNA immunoprecipitation (RIP) assays were performed. circSPIRE1 exhibited only weak binding affinity with Argonaute 2 (AGO2), indicating that circSPIRE1 is unlikely to exert its biological effects in prostate cancer through the miRNA sponge mechanism (Fig. 3A). Subsequently, sucrose gradient centrifugation was used to analyze circSPIRE1’s association with ribosomal complexes. Upon circSPIRE1 overexpression, a significant increase in circSPIRE1 was detected in the polysome fractions, indicating its active involvement in translation (Fig. 3B and Fig. S3A-B). Further analysis of circSPIRE1 binding to ribosomal subunits demonstrated that a substantial proportion of circSPIRE1 was associated with polysomes rather than monosomes, providing additional evidence for its role in translation regulation (Fig. 3C). Collectively, these findings strongly suggest that circSPIRE1 primarily functions through protein translation, rather than serving as a miRNA sponge.

Analysis of the TransCirc database revealed that circSPIRE1 contains an ORF with an initiation codon (ATG) but lacks a termination codon. Notably, circSPIRE1 is 369 nucleotides in length—a multiple of three—consistent with the requirements for continuous, frame-preserving translation. This structural configuration strongly suggests that circSPIRE1 is capable of rolling circle translation, potentially generating repeated polypeptide sequences (Fig. 3D and Fig. S;3C). To validate the translational capacity of circSPIRE1, three constructs were designed: (1) an empty vector as a control, (2) circSPIRE1-flag, which included a 3XFlag tag inserted immediately upstream of the start codon (ATG) at the end of the ORF loop, and (3) circSPIRE1-flag-mut, a modified version of the second construct with a cytosine (C) to thymine (T) mutation within 20 nucleotides downstream of the ATG, introducing a TAA stop codon. This mutation was designed to terminate translation while minimally disrupting the RNA secondary structure (Fig. 3E). This design enabled the detection of encoded protein products via the Flag tag. Western blot analysis revealed three main protein bands exclusively in the circSPIRE1-flag group, each with molecular weights in multiples of approximately 17 kDa—the calculated molecular weight for a single loop of the ORF product (Fig. 3F). To confirm that these bands represented circSPIRE1-encoded products, electrophoresis, silver staining, and mass spectrometry were performed, identifying specific peptide sequences encoded by circSPIRE1 (Fig. 3G and Fig. S3D). We designated the circSPIRE1-encoded products as “rtSPIRE1”, with molecular weights calculated as *N*×17 kDa, where N represents the number of translation loops.

The termination of rolling circle translation in circSPIRE1 may be mediated by programmed − 1 ribosomal frameshifting, a known mechanism for translation termination [16, 17, 23, 24]. To test whether circSPIRE1 encodes repeating units of its ORF, we generated linear expression vectors containing 1, 2, 3, and 4 repeats of the ORF, designated as 1R, 2R, 3R, and 4R, respectively (Fig. 3H). Western blot analysis demonstrated that the primary translation products of the 1R, 2R, and 3R constructs matched the major translation products of circSPIRE1, indicating that circSPIRE1 predominantly encodes proteins composed of up to three ORF repeats (Fig. 3I). In contrast, the 4R construct yielded minimal translation products, suggesting that circSPIRE1 rarely generates proteins with more than three ORF repeats. Additionally, faint bands corresponding to 1- and 2-repeat products were observed in the 3R and 4R constructs, likely resulting from programmed ribosomal frameshifting, which may induce premature translation termination and produce truncated polypeptides.

### hnRNPA1 regulates circSPIRE1 translation via modulation of IRES activity

To assess the predicted IRES activity within circSPIRE1, we constructed dual-luciferase reporter plasmids (Fig. 3J). The full-length circSPIRE1 IRES exhibited the highest Luc/Rluc activity, while deletion of the latter portion of the IRES sequence significantly reduced activity (Fig. 3K), indicating that circSPIRE1 drives translation through a cap-independent mechanism mediated by IRES.

**Fig. 4:**
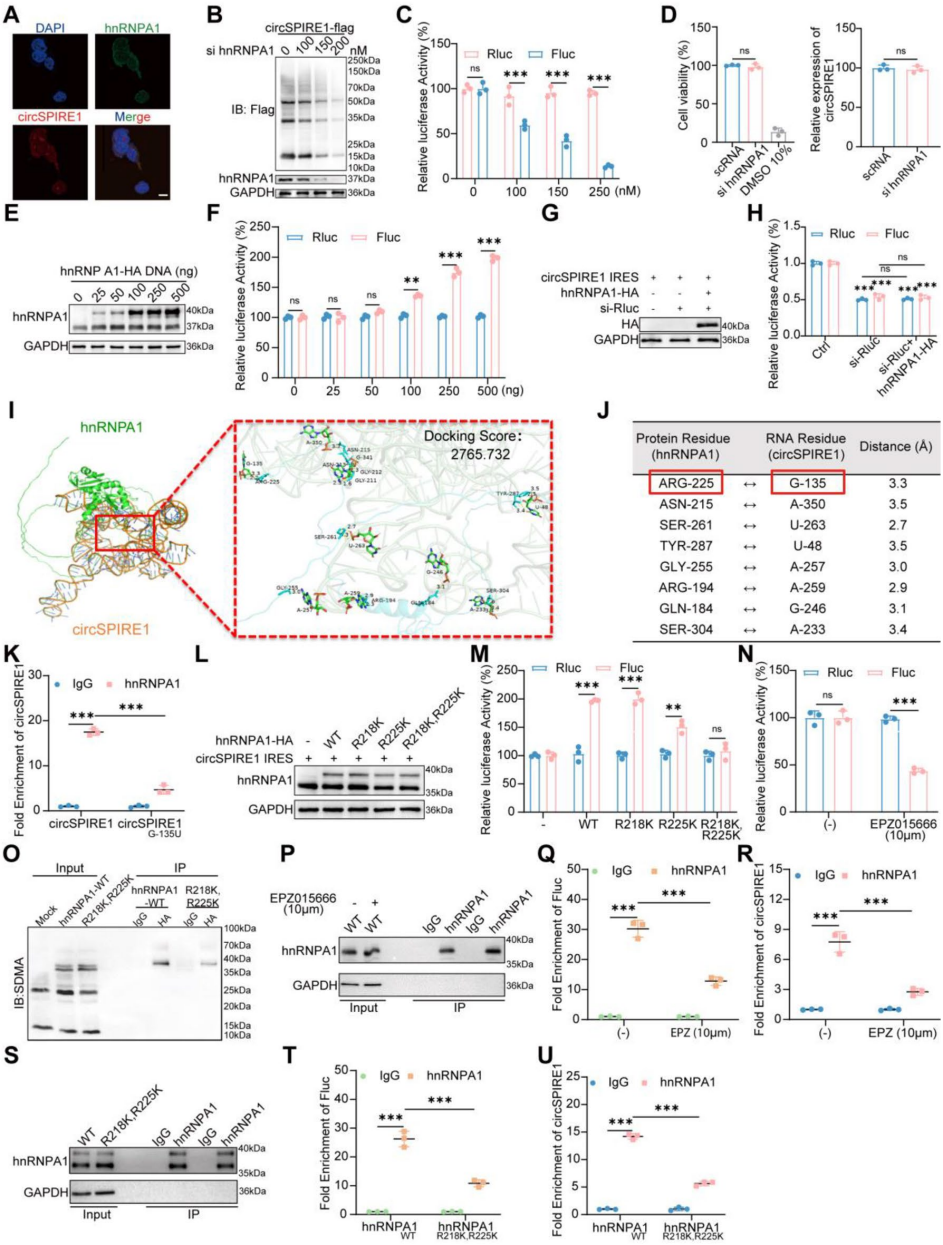
hnRNPA1 SDMA modification promotes circSPIRE1 IRES activity (**A**) FISH combined with immunofluorescence revealed cytoplasmic co-localization of hnRNPA1 and circSPIRE1 (scale bar = 10 μm; *n* = 3). (**B**) Western blot analysis showed hnRNPA1 knockdown reduced circSPIRE1 translation in a dose-dependent manner (*n* = 3). (**C**) Dual-luciferase assays demonstrated a dose-dependent decrease in Fluc activity upon hnRNPA1 knockdown, with no change in Rluc (*n* = 3). (**D**) CCK8 and RT-qPCR assays confirmed that short-term hnRNPA1 knockdown (150nM) did not significantly affect cell viability or circSPIRE1 RNA levels. (**E**) Western blot analysis showed a dose-dependent increase in exogenous hnRNPA1-HA expression (*n* = 3). (**F**) Fluc activity in circSPIRE1 IRES-transfected cells increased with hnRNPA1-HA expression, while Rluc activity remained unchanged (*n* = 3). (**G**-**H**) Rluc knockdown (50nM) validated the reliability of the dual-luciferase reporter (100ng) for assessing circSPIRE1 IRES activity. (**I**-**J**) Molecular docking identified G-135 as the binding site between circSPIRE1 and hnRNPA1. (**K**) RIP assay showed hnRNPA1 binding to wild-type circSPIRE1, but reduced binding to G135U-mutated circSPIRE1 (*n* = 3). (**L**-**M**) Schematic diagrams and luciferase assays of co-transfected constructs in 293T cells showing dose-dependent effects on Fluc and Rluc (*n* = 3). (**N**) SDMA inhibitor EPZ015666 (10 µM) reduced Fluc activity in circSPIRE1 IRES-transfected cells, with no effect on Rluc (*n* = 3). (**O**) IP assays revealed that the dual R218K/R225K mutation markedly reduced SDMA modification. (**P**-**R**) RIP assays following EPZ015666 treatment demonstrated a significant reduction in hnRNPA1 binding to both Fluc and circSPIRE1 (*n* = 3). (**S**-**U**) RIP assays using wild-type and mutant hnRNPA1 constructs showed that mutations at R218 and R225 significantly reduced hnRNPA1 binding to both Fluc and circSPIRE1 (*n* = 3). ns, no significance; *p < 0.05, **p < 0.01, ***p < 0.001

To identify potential ITAFs regulating circSPIRE1 IRES activity, we performed an RNA pulldown assay using a circSPIRE1-specific probe (Fig. 3L). Silver staining and mass spectrometry identified hnRNPA1 as an ITAF interacting with circSPIRE1 (Fig. S3E). The interaction was further validated through RNA pulldown assays using both cell lysates and recombinant hnRNPA1 protein, confirming hnRNPA1’s selective binding to circSPIRE1 (Fig. 3M-N). RIP assays revealed significant enrichment of circSPIRE1 in hnRNPA1-IP fractions compared to the U1 control (Fig. 3O). Additionally, RIP assays comparing wild-type circSPIRE1 with an IRES-deleted mutant (circSPIRE1ΔIRES) demonstrated that only the wild-type retained strong hnRNPA1 binding, while the mutant showed markedly reduced interaction (Fig. 3P), highlighting the IRES as a critical region for hnRNPA1-circSPIRE1 association. FISH combined with immunofluorescence confirmed significant cytoplasmic co-localization of hnRNPA1 and circSPIRE1 (Fig. 4A).

To elucidate hnRNPA1’s role in circSPIRE1 translation, we transfected prostate cancer cells with the dual-luciferase reporter construct containing the circSPIRE1 IRES. Western blot analysis showed that hnRNPA1 knockdown reduced circSPIRE1’s translational efficiency (Fig. 4B). Gradient knockdown of hnRNPA1 led to a dose-dependent decrease in Fluc activity (Fig. 4C). Importantly, short-term hnRNPA1 knockdown did not affect cell viability or circSPIRE1 RNA levels, confirming that the observed effects were specific to translation rather than RNA stability or cell health (Fig. 4D). Overexpression of hnRNPA1 increased Fluc activity without affecting Rluc activity (Fig. 4E-F), indicating that hnRNPA1 promotes circSPIRE1 IRES activity. These findings establish hnRNPA1 as a crucial ITAF, mediating circSPIRE1-driven rolling circle translation through IRES-dependent interactions in the cytoplasm.

To ensure accurate measurement of IRES activity without interference from potential monocistronic transcripts of Rluc or Fluc generated during circSPIRE1 IRES vector expression, we specifically knocked down Rluc (Fig. 4G). Consistent changes in both Rluc and Fluc levels confirmed that their parallel alterations reliably reflect IRES activity. Furthermore, changes in hnRNPA1 expression did not affect this consistency, reinforcing that hnRNPA1 expression changes do not interfere with the accurate measurement of IRES activity (Fig. 4H).

### Post-translational modifications of hnRNPA1 regulate circSPIRE1 IRES activity

To elucidate the mechanism by which hnRNPA1 regulates IRES activity, we first identified the interaction between hnRNPA1 and the IRES region of circSPIRE1. Molecular docking analysis revealed that G-135 within the latter half of the circSPIRE1 IRES region serves as the primary binding site for hnRNPA1 (Figs. 3K and 4I-J). Mutation of G-135 significantly impaired hnRNPA1 binding to circSPIRE1 (Fig. 4K), confirming the critical role of this site in the interaction. Previous studies have highlighted the importance of post-translational modifications at ARG-225 in hnRNPA1 for regulating IRES activity [20]. Additionally, symmetric dimethylation (SDMA) of ARG-225 and ARG-218 has been implicated in modulating IRES function [20, 21]. Based on these findings, we hypothesized that hnRNPA1 regulates circSPIRE1 IRES activity through a similar PTM-dependent mechanism.

To test this hypothesis, we transfected 293T cells with the circSPIRE1 IRES reporter construct along with either wild-type hnRNPA1 (WT) or mutants at residues R218 and R225 (R218K, R225K, or R218K, R225K) (Fig. 4L). Luciferase assays showed that the R225K mutation reduced IRES activity, while the R218K mutation had no significant effect. Notably, the double mutation (R218K, R225K) caused a marked suppression of IRES activity, suggesting a synergistic role of these residues in regulating circSPIRE1 translation (Fig. 4M).

To further investigate the role of SDMA modifications, we treated cells with the SDMA inhibitor EPZ015666. This treatment significantly reduced circSPIRE1 IRES activity (Fig. 4N). Immunoprecipitation assays revealed that the SDMA modification level of hnRNPA1 was markedly decreased in the R218K, R225K mutant, indicating that these mutations impair SDMA modification (Fig. 4O). These results suggest that SDMA of hnRNPA1 is essential for regulating circSPIRE1 translation via the IRES mechanism.

To confirm the functional importance of SDMA modifications, we performed RIP assays in cells treated with EPZ015666. The inhibitor significantly reduced hnRNPA1 binding to both Fluc and circSPIRE1, demonstrating that SDMA modifications are critical for hnRNPA1’s interaction with the IRES element (Fig. 4P-R). Additionally, RIP assays with hnRNPA1 mutants (R218K, R225K, and R218K, R225K) showed that mutations at these residues significantly diminished hnRNPA1’s binding affinity to Fluc and circSPIRE1, further supporting the essential roles of ARG-218 and ARG-225 in this regulatory pathway (Fig. 4S-U).

Collectively, these findings reveal that SDMA-dependent modifications of hnRNPA1 modulate its binding to circSPIRE1, thereby regulating IRES activity and driving circSPIRE1 translation. This study uncovers a novel mechanism by which specific PTMs of hnRNPA1 influence circRNA-mediated protein synthesis, highlighting the functional significance of hnRNPA1 modifications in circSPIRE1 biology.

### Clinical relevance of the novel protein rtSPIRE1 in prostate cancer

**Fig. 5:**
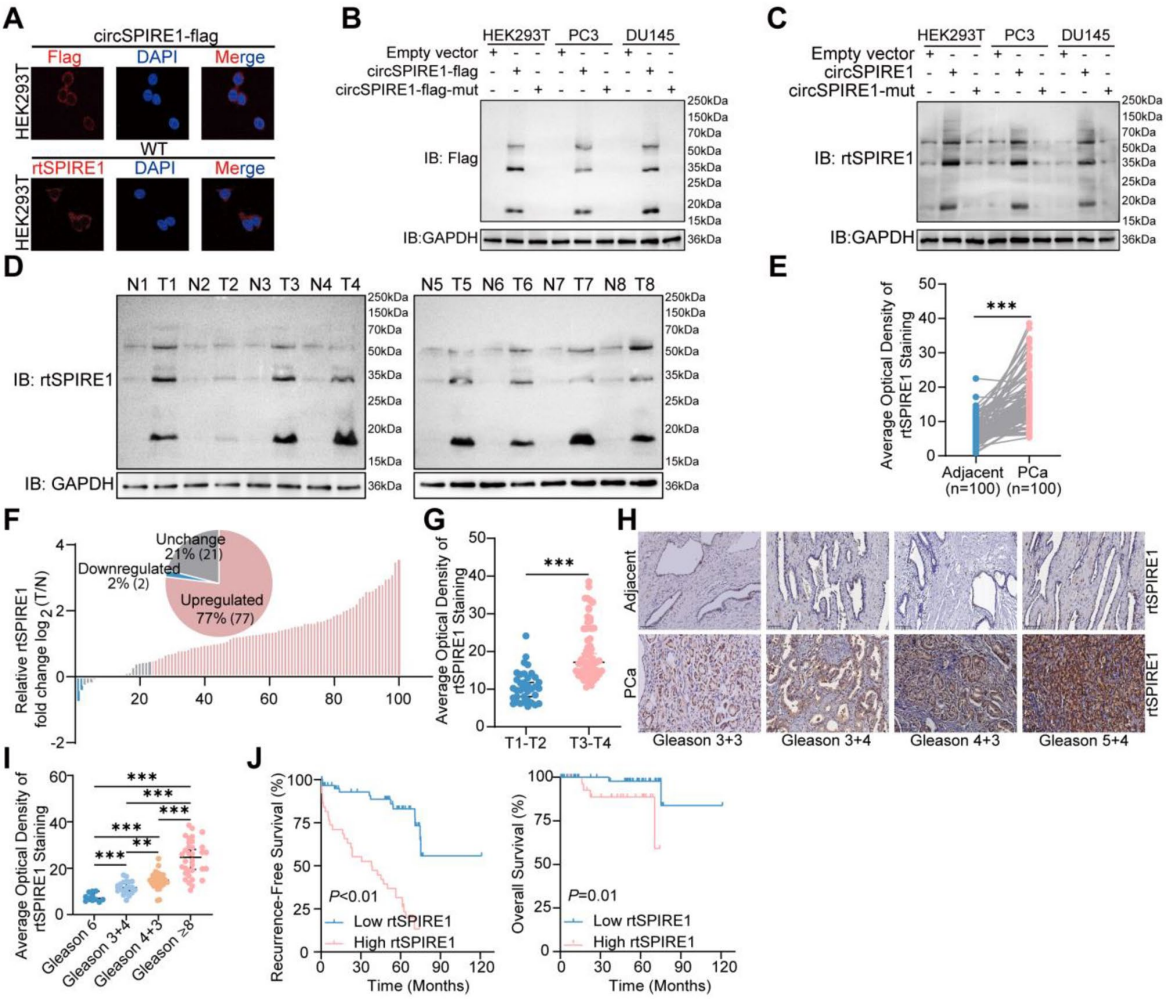
Clinical Relevance of the Novel Protein rtSPIRE1 in Prostate Cancer (**A**) Immunofluorescence analysis revealed the cytoplasmic localization of rtSPIRE1. Staining with a custom rtSPIRE1 antibody exhibited a pattern consistent with Flag antibody staining, confirming specific recognition (scale bar = 10 μm; *n* = 3). (**B**) Western blot analysis detected overexpressed rtSPIRE1 using the Flag antibody. (**C**) The custom rtSPIRE1 antibody identified both overexpressed and endogenous rtSPIRE1, confirming its specificity and sensitivity. (**D**) Western blot analysis of rtSPIRE1 expression in 8 paired prostate cancer (T) and adjacent non-cancerous (N) tissues revealed elevated rtSPIRE1 levels in most prostate cancer samples. (**E**) Expression levels of rtSPIRE1 in prostate cancer (PCa) tissues were assessed by immunohistochemical staining (*n* = 100). (**F**) Relative expression changes of rtSPIRE1 in PCa tissues (T) compared to paired normal tissues (N) were calculated as calculated as log2 fold change (T/N). (**G**) The average optical density of rtSPIRE1 staining was compared between PCa tissues at different stages (T1-T2 vs. T3-T4). (**H**) Representative immunohistochemical staining of rtSPIRE1 in normal prostate tissues (Adjacent) and PCa tissues with varying Gleason scores (Gleason 3 + 3, 3 + 4, 4 + 3, 5 + 4). (**I)** Quantification of rtSPIRE1 average optical density in PCa tissues stratified by Gleason scores. (**J**) High rtSPIRE1 expression was associated with reduced recurrence-free survival and overall survival in PCa patients (Kaplan-Meier survival curve). ns, no significance; *p < 0.05, **p < 0.01, ***p < 0.001

To validate the specificity and effectiveness of a custom rabbit polyclonal antibody against the unique rtSPIRE1 peptide sequence (RKVMLCAAHLPTES), we performed immunofluorescence and Western blot analyses. Immunofluorescence results demonstrated that the rtSPIRE1 antibody specifically recognized the cytoplasmic localization of rtSPIRE1, exhibiting a staining pattern consistent with that observed using the Flag antibody in overexpression systems (Fig. 5A). In Western blot assays, the rtSPIRE1 antibody not only detected overexpressed rtSPIRE1 bands, which aligned with those identified by the Flag antibody but also successfully recognized endogenous rtSPIRE1 protein bands. These findings confirm the high sensitivity and specificity of the custom antibody for rtSPIRE1 under both endogenous and overexpression conditions (Fig. 5B-C).

To investigate the clinical relevance of rtSPIRE1, we performed Western blot analysis on protein samples from eight matched pairs of prostate cancer and adjacent non-cancerous tissues (Fig. 5D). Furthermore, immunohistochemical staining of rtSPIRE1 in a cohort of 100 matched prostate cancer and adjacent non-cancerous tissue samples confirmed its specific overexpression in malignant tissues (Fig. 5E-F). Notably, rtSPIRE1 expression was significantly higher in T3-T4 stage tumors compared to T1-T2 stage tumors (Fig. 5G). Quantitative analysis demonstrated a positive correlation between rtSPIRE1 expression levels and prostate cancer malignancy grade, as assessed by the Gleason score (Fig. 5H-I). Detailed associations between rtSPIRE1 expression levels and clinicopathological parameters are summarized in Table S5. Additionally, higher rtSPIRE1 expression was associated with shorter recurrence-free survival and overall survival (Fig. 5J). These findings suggest that rtSPIRE1 may serve as a potential biomarker for prostate cancer progression and prognosis.

### The novel protein rtSPIRE1 promotes prostate cancer proliferation and metastasis

**Fig. 6:**
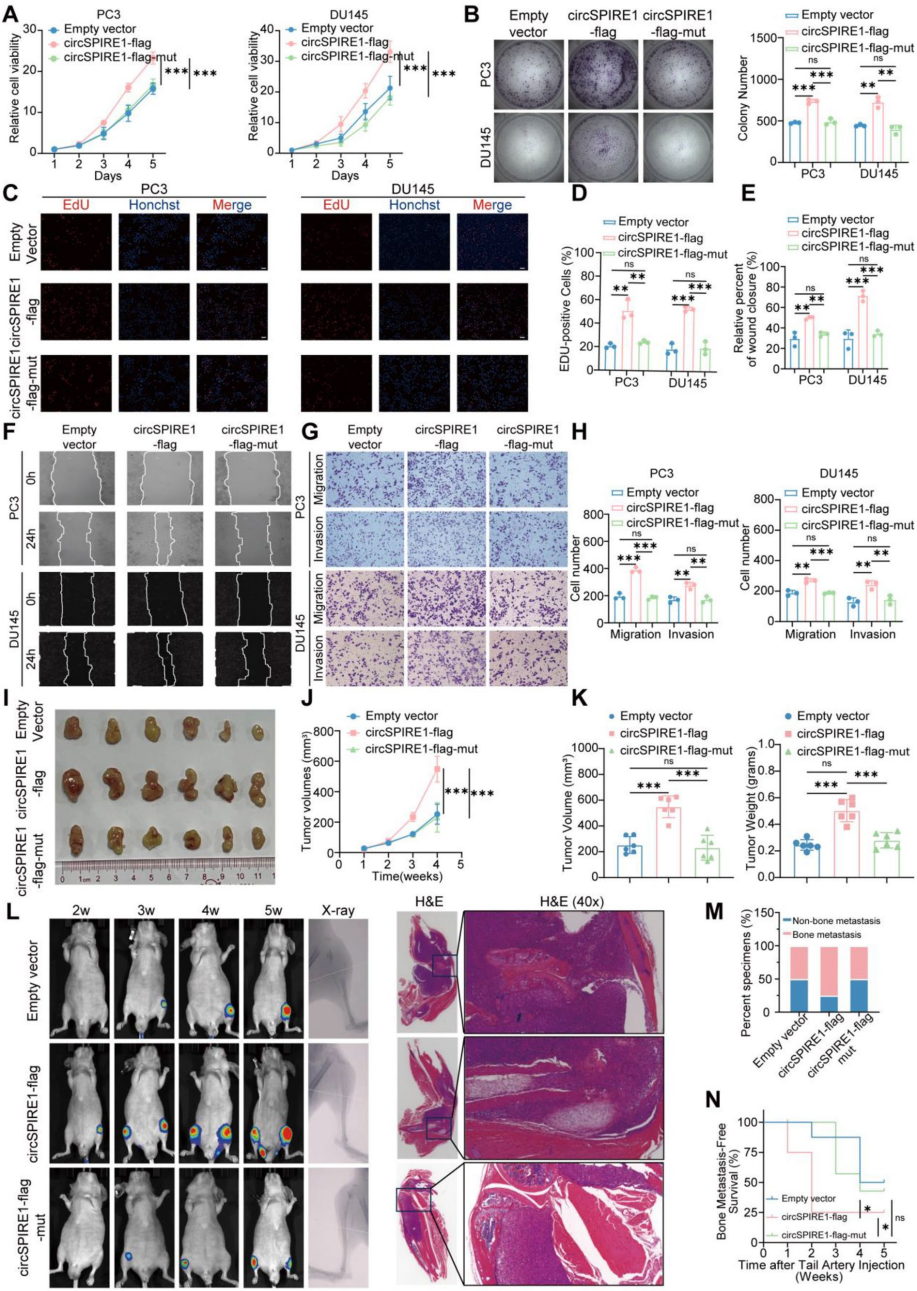
The Novel Protein rtSPIRE1 Promotes Prostate Cancer Proliferation and Metastasis (**A**) CCK8 assay results showing enhanced proliferation in the circSPIRE1-flag group compared to the Empty vector and circSPIRE1-flag-mut groups (*n* = 3). (**B**) Colony formation assay results revealing increased colony formation in the circSPIRE1-flag group (*n* = 3). (**C**-**D**) EdU incorporation assay results indicating higher proliferation in the circSPIRE1-flag group (*n* = 3). (**E**-**F**) Wound healing assay results showing accelerated wound closure in the circSPIRE1-flag group (*n* = 3). (**G**-**H**) Transwell migration and invasion assay results demonstrating enhanced migration and invasion in the circSPIRE1-flag group (*n* = 3). (**I**-**K**) Subcutaneous xenograft proliferation model in BALB/c nude mice using the PC3 cell line, comparing tumor growth among Empty vector, circSPIRE1-flag (encoding rtSPIRE1), and circSPIRE1-flag-mut (non-encoding) groups. Tumors in the rtSPIRE1-encoding group displayed significantly increased growth, with larger tumor volumes and weights than those in the control groups, indicating enhanced proliferative capacity (*n* = 6). (**L**) Tail artery injection bone metastasis model in BALB/c nude mice, representative images of bioluminescent imaging, X-ray, and H&E staining in circSPIRE1-flag cells compared to the Empty vector and circSPIRE1-flag-mut groups. (**M**) Statistical analysis reveals a significantly increased rate of bone metastasis in the circSPIRE1-flag group (*n* = 8). (N) Kaplan-Meier survival analysis showing shorter overall survival in BALB/c nude mice within the circSPIRE1-flag group (*n* = 8). ns, no significance; *p < 0.05, **p < 0.01, ***p < 0.001

To further explore the biological function of rtSPIRE1, we conducted a series of functional assays, including CCK8 (Fig. 6A), colony formation (Fig. 6B), EdU incorporation (Fig. 6C-D), wound healing (Fig. 6E-F), and Transwell migration and invasion (Fig. 6G-H). These experiments were performed across three groups: circSPIRE1-flag (capable of encoding rtSPIRE1), circSPIRE1-flag-mut (unable to encode rtSPIRE1 but retaining the RNA structure), and empty vector control. The circSPIRE1-flag group exhibited significantly enhanced proliferation, invasion, and migration capabilities compared to both the empty vector and circSPIRE1-flag-mut groups. These results indicate that rtSPIRE1 plays a critical role in promoting tumor progression.

To validate the function of rtSPIRE1 in vivo, we established a subcutaneous xenograft model using the same three groups: empty vector, circSPIRE1-flag, and circSPIRE1-flag-mut. In this model, tumors derived from the rtSPIRE1-encoding group displayed significantly accelerated growth, as evidenced by larger tumor volumes and weights (Fig. 6I-K). Histological analysis revealed pronounced differences, with the circSPIRE1-flag group showing elevated expression of Ki-67, N-cadherin, and vimentin, alongside reduced E-cadherin levels, suggesting enhanced proliferation and EMT characteristics (Fig. S3F).

Additionally, in a bone metastasis model established through tail artery injection, the rtSPIRE1-encoding group exhibited a significantly higher incidence of bone metastatic lesions than the other groups. This was confirmed by bioluminescent imaging, X-ray analysis, and H&E staining (Fig. 6L-M). Survival analysis further revealed that mice in the rtSPIRE1-encoding group had significantly shorter overall survival compared to the other groups (Fig. 6N). These results highlight the critical role of rtSPIRE1 in promoting prostate cancer aggressiveness and metastatic potential. Collectively, these findings underscore the significant impact of rtSPIRE1 in accelerating tumor growth and facilitating metastatic progression, particularly in the formation of bone metastases.

### rtSPIRE1 activates PI3K/AKT signaling by inhibiting LRP5 ubiquitination

**Fig. 7:**
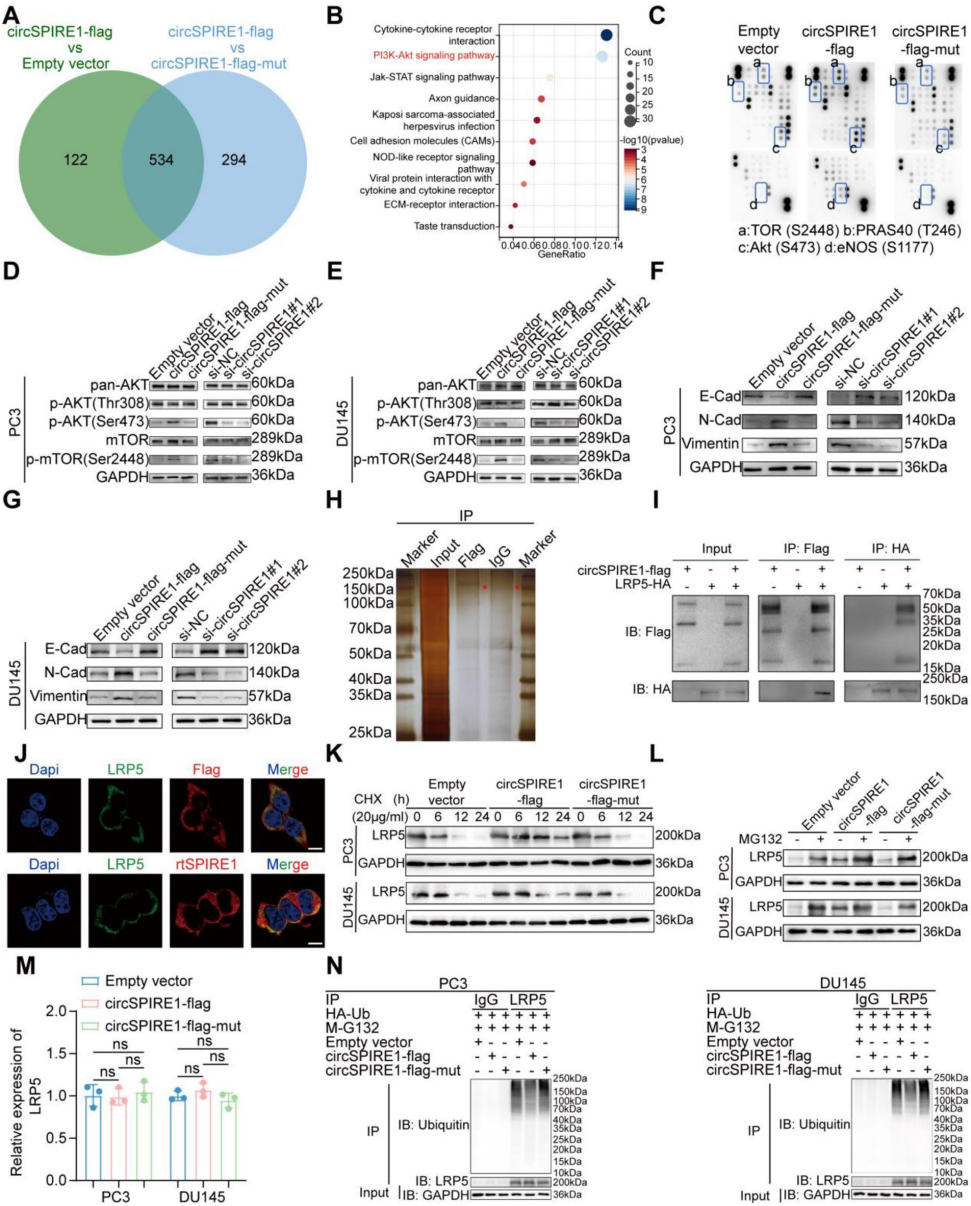
circSPIRE1 activates the PI3K/AKT signaling pathway and stabilizes LRP5 by inhibiting its ubiquitination (**A**) Venn diagram showing the overlap of differentially expressed genes (DEGs) between the circSPIRE1-flag vs. Empty vector and circSPIRE1-flag vs. circSPIRE1-flag-mut groups, identifying 534 shared genes. (**B**) KEGG pathway enrichment analysis of the intersecting genes highlights the PI3K/AKT signaling pathway as one of the significantly enriched pathways (*n* = 3). (**C**) Human phosphokinase array reveals elevated phosphorylation of p-mTOR (S2448) and p-AKT (S473) in the circSPIRE1-flag group (*n* = 3). (**D**-**E**) Western blot analysis in PC3 and DU145 cells shows increased phosphorylation of AKT (Ser473) and mTOR (Ser2448) in the circSPIRE1-flag group, while circSPIRE1 knockdown reduces phosphorylation levels (*n* = 3). (**F**-**G**) EMT marker analysis in PC3 and DU145 cells demonstrates elevated levels of N-cadherin and vimentin, along with reduced E-cadherin, in the circSPIRE1-flag group. Knockdown of circSPIRE1 reverses this pattern, indicating that circSPIRE1 promotes an EMT phenotype in prostate cancer cells (*n* = 3). (**H**) Immunoprecipitation and mass spectrometry identify LRP5 as a key interaction partner of circSPIRE1 (see Fig. S4G for detailed mass spectrum; *n* = 3). (I) Co-immunoprecipitation confirms the interaction between circSPIRE1 and LRP5 (*n* = 3). (**J**) Immunofluorescence imaging shows cytoplasmic co-localization of circSPIRE1 and LRP5 in cells (scale bar = 10 μm; *n* = 3). (**K**) Cycloheximide treatment demonstrates that circSPIRE1 prolongs LRP5’s half-life by reducing its degradation rate (*n* = 3). (**L**) Western blot analysis after MG132 treatment reveals elevated LRP5 protein levels in the circSPIRE1-flag group (*n* = 3). (**M**) RT-qPCR analysis confirms that circSPIRE1 does not affect LRP5 mRNA levels (*n* = 3). (**N**) Ubiquitination assays indicate that circSPIRE1 decreases LRP5 ubiquitination levels (*n* = 3). ns, not significant; *p < 0.05, **p < 0.01, ***p < 0.001

To investigate the signaling mechanisms by which rtSPIRE1 promotes prostate cancer proliferation and metastasis, we conducted transcriptomic sequencing, comparing the circSPIRE1-flag group with the Empty vector and circSPIRE1-flag-mut groups. Functional enrichment analyses independently identified the PI3K/AKT signaling pathway as a top candidate in both comparisons (Fig. S4A-F). Intersection analysis of differentially expressed genes from these comparisons (Fig. 7A-B) and subsequent enrichment analysis further confirmed the prominence of the PI3K/AKT pathway, suggesting that rtSPIRE1 drives prostate cancer progression through its activation.

To validate these findings, we employed a human phosphokinase array and observed that rtSPIRE1 expression was associated with significant activation of key proteins within the PI3K/AKT signaling pathway. Specifically, phosphorylation levels of p-mTOR (S2448) and p-AKT (S473) were markedly elevated in the rtSPIRE1-expressing group (Fig. 7C). These results strongly support the hypothesis that rtSPIRE1 enhances oncogenic signaling by activating the PI3K/AKT pathway.

To study the role of rtSPIRE1 in regulating the AKT/mTOR pathway, we performed Western blot assays using PC3 and DU145 cell models. The results revealed significantly increased phosphorylation of AKT (Ser473) and mTOR (Ser2448) in the circSPIRE1-flag group compared to the empty vector and circSPIRE1-flag-mut groups, while circSPIRE1 knockdown markedly reduced these phosphorylation levels (Fig. 7D-E). Additionally, the circSPIRE1-flag group exhibited elevated expression of N-cadherin and vimentin, along with reduced E-cadherin levels, indicating an EMT shift. Conversely, circSPIRE1 knockdown reversed these changes, with decreased N-cadherin and vimentin and increased E-cadherin expression (Fig. 7F-G). These findings collectively suggest that rtSPIRE1 promotes prostate cancer cell proliferation and migration by activating the PI3K/AKT/mTOR pathway and facilitating EMT.

To elucidate the mechanism by which rtSPIRE1 activates the PI3K/AKT pathway, we performed IP and mass spectrometry analyses, identifying LRP5 as a key interacting partner of rtSPIRE1 (Fig. 7H and Fig. S4G). Given that LRP5 has been reported to activate the PI3K/AKT pathway [25–28], we hypothesized that rtSPIRE1 mediates PI3K/AKT activation through LRP5. Co-IP experiments confirmed the physical interaction between rtSPIRE1 and LRP5 (Fig. 7I), and immunofluorescence analysis revealed strong cytoplasmic co-localization of the two proteins (Fig. 7J). Cycloheximide treatment demonstrated that rtSPIRE1 prolonged LRP5’s half-life by slowing its degradation rate (Fig. 7K). To further assess LRP5 stability, we treated cells with the proteasome inhibitor MG132 and observed higher LRP5 protein levels in the circSPIRE1-flag group, indicating that rtSPIRE1 inhibits LRP5 degradation (Fig. 7L). Notably, rtSPIRE1 did not affect LRP5 mRNA levels, suggesting a post-translational regulatory mechanism (Fig. 7M). Ubiquitination assays revealed reduced LRP5 ubiquitination in the presence of rtSPIRE1, supporting the conclusion that rtSPIRE1 stabilizes LRP5 by inhibiting its ubiquitination, thereby enhancing PI3K/AKT pathway activation (Fig. 7N).

**Fig. 8:**
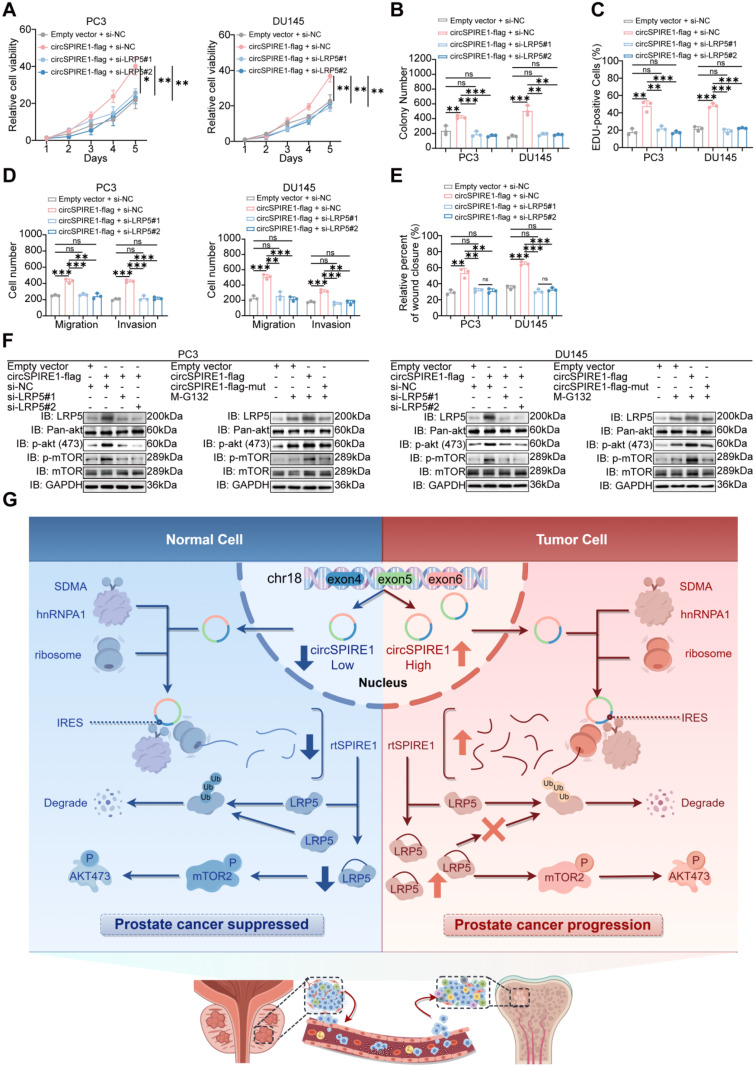
LRP5 is essential for rtSPIRE1-mediated proliferation, migration, and PI3K/AKT activation (**A**) CCK8 assay shows that silencing LRP5 significantly reduces rtSPIRE1-induced cell viability (*n* = 3; see Fig. S5A for representative images). (**B**) Colony formation assay reveals that LRP5 knockdown decreases rtSPIRE1-mediated colony-forming ability (*n* = 3; see Fig. S5A for representative images). (**C**) EdU incorporation assay demonstrates that LRP5 silencing suppresses rtSPIRE1-driven cell proliferation (*n* = 3; see Fig. S5B for representative images). (**D**) Transwell migration and invasion assays indicate that LRP5 knockdown attenuates rtSPIRE1-induced cell migration and invasion (*n* = 3; see Fig. S5C for representative images). (**E**) Wound healing assay confirms that LRP5 silencing impairs rtSPIRE1-promoted cell motility (*n* = 3; see Fig. S5C for representative images). (**F**) Western blot analysis shows that LRP5 knockdown (using siLRP5#1 and siLRP5#2) in circSPIRE1-flag-overexpressing cells reduces phosphorylation levels of p-AKT (Ser473) and p-mTOR (Ser2448), aligning them closer to baseline levels observed in the empty vector and circSPIRE1-flag-mut groups. Treatment with MG132 partially restores these phosphorylation levels (*n* = 3). (**G**) Schematic illustration of hnRNPA1-mediated rolling circle translation of circSPIRE1 promoting prostate cancer progression via the PI3K/AKT pathway. ns, not significant; *p < 0.05, **p < 0.01, ***p < 0.001

To confirm that LRP5 is a critical mediator of the pro-tumorigenic effects of rtSPIRE1 in prostate cancer, we conducted a series of rescue experiments. These included CCK8, colony formation, EdU, Transwell, and wound healing assays, which demonstrated that LRP5 knockdown significantly attenuated the enhanced proliferation, migration, and invasion induced by rtSPIRE1 overexpression (Fig. 8A-E and Fig. S5A-C). Western blot analysis further validated this role, showing that LRP5 knockdown (using siLRP5#1 and siLRP5#2) in rtSPIRE1-overexpressing cells markedly reduced levels of p-AKT (Ser473) and p-mTOR (Ser2448), bringing them closer to baseline levels observed in the empty vector and circSPIRE1-flag-mut groups (Fig. 8F). Treatment with the proteasome inhibitor MG132 partially restored these phosphorylated protein levels, suggesting that rtSPIRE1 activates the PI3K/AKT signaling pathway by stabilizing LRP5 through inhibition of its ubiquitination. Collectively, these findings confirm that LRP5 is a key mediator in rtSPIRE1-induced activation of the PI3K/AKT signaling pathway.

Our study revealed that circSPIRE1, regulated by hnRNPA1’s SDMA-mediated post-translational modifications, undergoes rolling circle translation to encode rtSPIRE1, which promotes prostate cancer progression by stabilizing LRP5 and activating the PI3K/AKT signaling pathway. Specifically, rtSPIRE1 enhances LRP5 stability by inhibiting its ubiquitination, leading to increased phosphorylation and activation of downstream PI3K/AKT signaling. This pathway activation drives enhanced cell proliferation, migration, and invasion in prostate cancer (Fig. 8G).

## Discussion

In this study, we investigated the expression and functional roles of circSPIRE1 and its encoded peptide rtSPIRE1, demonstrating their contribution to prostate tumor progression through rolling circle translation. First, both circSPIRE1 and rtSPIRE1 were markedly elevated in prostate cancer tissues and cell lines, and rtSPIRE1 significantly enhanced cancer cell proliferation, migration, and invasion, all of which contribute to tumor progression. Second, we demonstrated that the rolling circle translation of circSPIRE1 is regulated by SDMA modifications of hnRNPA1, which modulate IRES activity in circSPIRE1. Finally, rtSPIRE1 stabilizes LRP5 by inhibiting its ubiquitination, activating the PI3K/AKT/mTOR signaling pathway to promote tumor progression. Together, these findings provide a new perspective on the oncogenic roles of circSPIRE1 and rtSPIRE1, suggesting potential therapeutic strategies targeting circRNAs, their interacting proteins, and encoded peptides in prostate cancer.

In this study, we identified circSPIRE1 as one of the most significantly altered and translationally active circRNAs in prostate cancer. Our data revealed that circSPIRE1 expression is highly specific to prostate cancer tissues, and its inhibition markedly reduces cancer cell proliferation and migration. Additionally, we examined the coding potential of circSPIRE1 and identified a 369-nt ORF encoding a unique peptide, rtSPIRE1, through IRES-driven rolling circle translation. Importantly, this IRES activity is regulated by hnRNPA1, whose SDMA-mediated post-translational modifications enhance circSPIRE1 translation. Our findings demonstrated that rtSPIRE1 enhances oncogenic signaling in prostate cancer by stabilizing LRP5, activating the PI3K/AKT pathway and promoting cancer progression.

Previous studies have demonstrated the diverse roles of circRNAs, including their interactions with microRNAs and RNA-binding proteins [29]. Many circRNAs can also encode functional peptides that modulate signaling pathways, tumor immunity, apoptosis, and cell cycle progression [12, 30, 31]. These translational processes are often IRES-dependent, facilitating ribosome recruitment and protein synthesis [15, 32]. Notable examples include circ-EIF6, encoding the peptide EIF6-224aa31 through IRES-driven translation [33], which interacts with MYH9 to activate the Wnt/β-catenin pathway, and circZKSCAN1, which enhances sorafenib sensitivity in liver cancer [34]. Yet, few studies have identified factors that directly regulate IRES-mediated ribosome recruitment. Here, we demonstrated that hnRNPA1 serves as a key ITAF that modulates the binding affinity to the IRES region of circSPIRE1, promoting ribosomal recruitment and efficient translation. Notably, the SDMA post-translational modification of hnRNPA1 differentially regulates this binding, highlighting its critical role in regulating circSPIRE1 translation.

While most research on circRNA-encoded proteins has focused on linear RNA-like translation mechanisms, rolling circle translation—particularly its termination—remains poorly understood. Our study reveals that circSPIRE1 translation terminates through − 1 programmed ribosomal frameshifting, a phenomenon rarely reported in prior circRNA studies [17]. Whether the functions of circRNAs in cancers are mediated through rolling circle translation remains largely uncertain. In the case of circSPIRE1, we demonstrated that it promotes prostate tumor progression, suggesting its potential as a therapeutic target. Our findings highlight the unique roles of rolling circle translation in cancers.

The PI3K plays a crucial role in driving tumor development and progression by promoting cell survival, proliferation, metastasis, and angiogenesis [35, 36]. For instance, the PI3K pathway promotes progression to castration-resistant prostate cancer following androgen deprivation therapy via interactions with the androgen receptor pathway [37, 38]. Additionally, abnormal activation of PI3K contributes to docetaxel resistance and can induce EMT or neuroendocrine transformation [39, 40]. However, clinical trials employing PI3K pathway inhibitors as monotherapies have shown limited efficacy in prostate cancer, likely owing to compensatory pathway activation [41, 42]. Our study provides new insights by revealing that rtSPIRE1 can stabilize LRP5, triggering mTOR and AKT phosphorylation. This finding deepens our insight into PI3K/AKT pathway regulation in prostate cancer.

We identify rtSPIRE1 as a novel binding partner of LRP5, providing new insights into LRP5-mediated signaling in tumor progression. Overexpression of LRP5 enhances the expression and activation of β-catenin and AKT in aggressive cancers [25, 26, 43]. Previous studies have demonstrated that Hsp90ab1 binds LRP5 to activate AKT and Wnt/β-catenin signaling, promoting invasion and metastasis in gastric cancer cells [28]. Similarly, LRP5 deficiency induces AKT degradation,, while JAM3 directly interacts with LRP5 to enhance PDK1/AKT signaling, supporting leukemic stem cell self-renewal [26]. In our study, we demonstrated that rtSPIRE1 reduces ubiquitin-mediated proteasomal degradation of LRP5, resulting in LRP5 upregulation within prostate cancer cells. Importantly, LRP5 knockdown attenuates rtSPIRE1-induced proliferation and invasion in prostate cancer, underscoring the pivotal role of the rtSPIRE1-LRP5 interaction in cancer progression. Together, these findings reveal that rtSPIRE1 critically regulates LRP5 stability and function, establishing a key mechanism through which rtSPIRE1 facilitates tumor growth and metastasis in prostate cancer.

This study provides novel insights into the roles of circSPIRE1 and rtSPIRE1, primarily through cell-based systems and initial in vivo validation using xenograft models. While these findings advance our understanding of rtSPIRE1’s involvement in PI3K/AKT pathway modulation and LRP5 interactions, further exploration using advanced animal models or tumor organoids could offer deeper mechanistic insights into its functions within the tumor microenvironment.

## Conclusions

In conclusion, Our study reveals that hnRNPA1 regulates the IRES-driven rolling circle translation of circSPIRE1, generating the functional peptide rtSPIRE1. By stabilizing LRP5, rtSPIRE1 activates the PI3K/AKT signaling pathway, thereby promoting prostate cancer progression. These findings highlight the therapeutic potential of targeting circRNA-encoded peptides for prostate cancer treatment.

## Electronic supplementary material

Below is the link to the electronic supplementary material.

Supplementary Material 1

## Data Availability

Data is provided within the manuscript or supplementary information files.

## Abbreviations

circRNAs: Circular RNAs
ADT: Androgen deprivation therapy
EMT: Epithelial-mesenchymal transition
IRES: Internal ribosome entry sites
m6A: N6-methyladenosine
IRES: Dependent trans-acting factors (IRES)
hnRNPA1: Heterogeneous nuclear ribonucleoprotein A1
ORF: Open reading frame
LRP5: LDL receptor-related protein 5
DEcRs: Differentially expressed circRNAs
IF: Immunofluorescence
cDNA: Complementary DNA
gDNA: Genomic DNA
FISH: Fluorescence in situ hybridization
RIP: RNA immunoprecipitation
AGO2: Argonaute 2
IP: Immunoprecipitation
