# Supplementary material for: A novel polypeptide encoded by circSPIRE1 promotes prostate cancer proliferation and migration by restraining the ubiquitin-dependent degradation of LRP5
Source: J Exp Clin Cancer Res. 2025 Jul 25;44:218. doi: 10.1186/s13046-025-03467-8 (PMC12291493; doi:10.1186/s13046-025-03467-8)
Supplement: Supplementary file 1 — Supplementary Material 1 [file 13046_2025_3467_MOESM1_ESM.docx]

**Supplementary Materials**

**A novel polypeptide encoded by circSPIRE1 promotes prostate cancer proliferation and migration by restraining the ubiquitin-dependent degradation of LRP5.**

Jintao Hu ^a b c^, Juanyi Shi ^a b c^, Junjie Wang ^a b c^, Yunfei Xiao ^a b c^, Degeng Kong ^a b c^, Mingchao Gao ^a b c^, Tianlong Luo ^a b c^, Shizhong Xu ^a b c^, Zhihan Yuan ^a b c^, Xinyi Ma ^d^, Xueseng Dong ^e^, Jingang Huang ^b *^, Cheng Liu ^a b c f *^, Kewei Xu ^a b c d *^

**This supplementary file includes:**

Supplementary Figures S1 to S6

Supplementary Tables S1 to S6


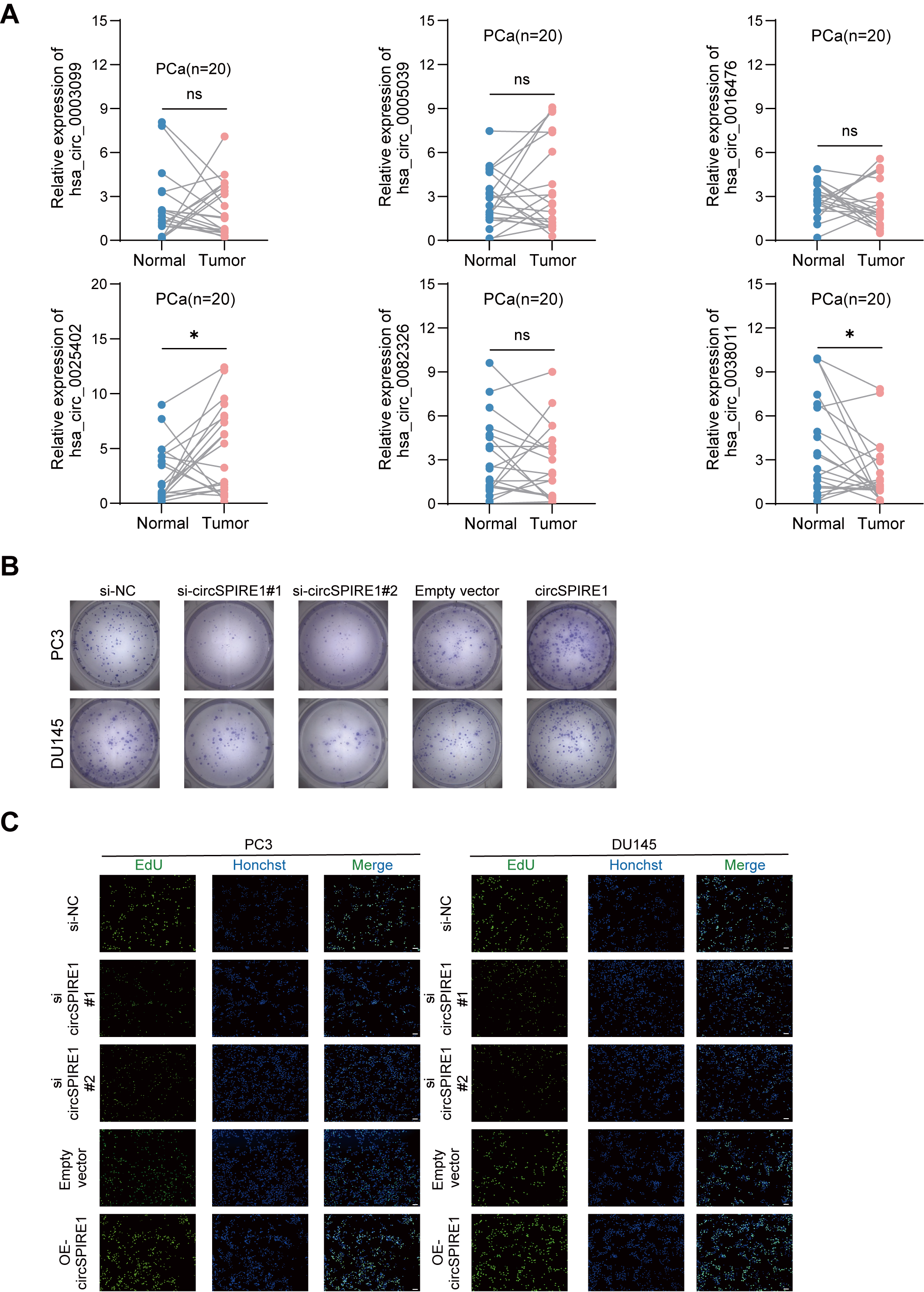


**Fig. S1 Validation and functional analysis of screened circRNAs**

1. RT-qPCR validation of six screened circRNAs (excluding circSPIRE1). (B) Representative images of colony formation assay after overexpression and knockdown of circSPIRE1. (C) Representative images of EdU incorporation assay after overexpression and knockdown of circSPIRE1.


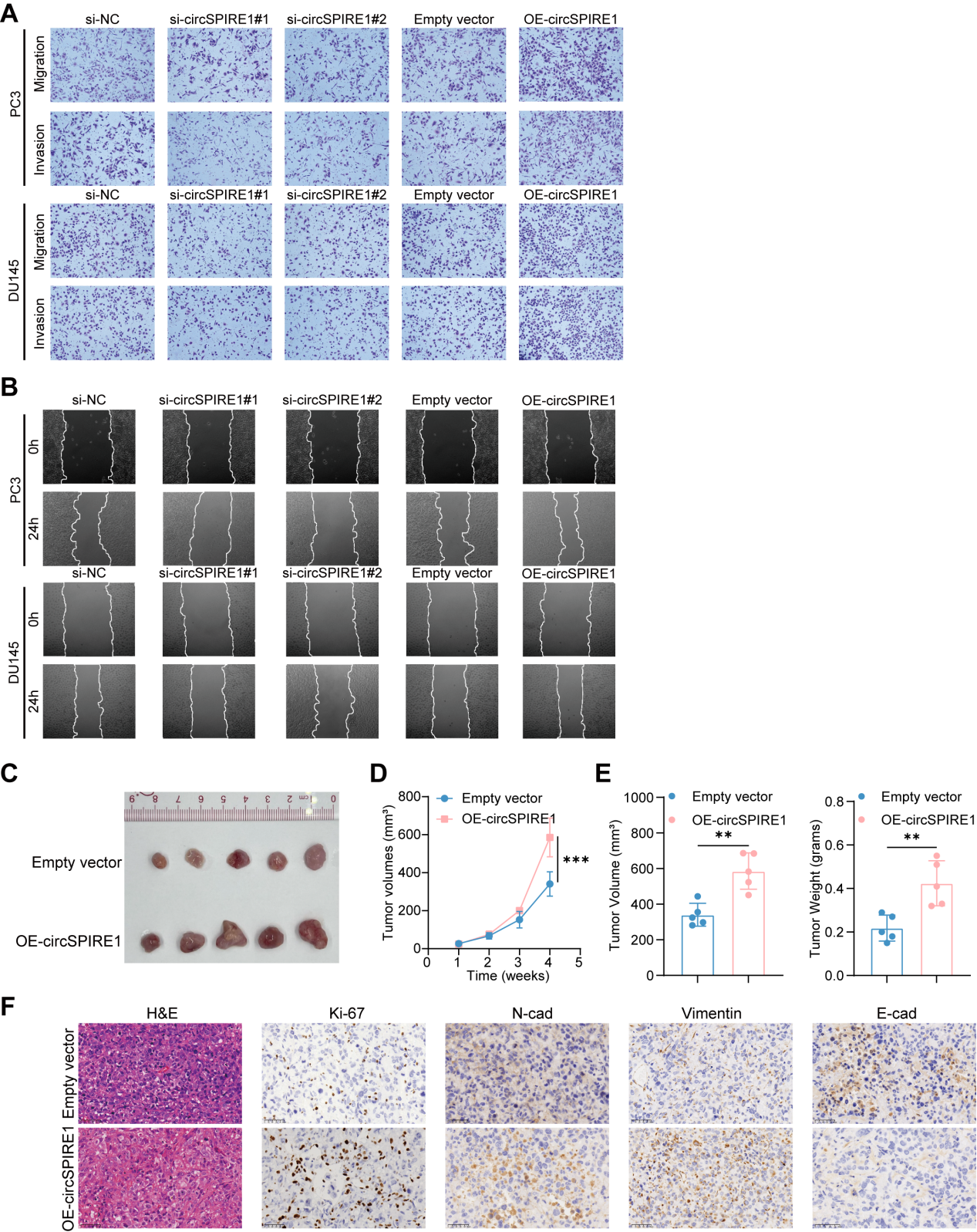


**Fig. S2 Functional analysis of circSPIRE1 in vitro and in vivo**

1. Representative images of Transwell assay after overexpression and knockdown of circSPIRE1. (B) Representative images of wound healing (scratch) assay after overexpression and knockdown of circSPIRE1. (C) In vivo xenograft experiments in BALB/c nude mice demonstrate that tumors derived from circSPIRE1-overexpressing cells exhibit significantly increased volume and weight compared to control groups, indicating enhanced tumor growth potential. (C-E) In vivo xenograft experiments in BALB/c nude mice demonstrating enhanced tumor growth potential in circSPIRE1-overexpressing cells compared to control groups. (F) Immunohistochemical staining of xenograft tumors reveals higher expression of the proliferation marker Ki-67 and mesenchymal markers N-cadherin and vimentin in circSPIRE1-overexpressing tumors, with reduced E-cadherin levels, suggesting increased tumor aggressiveness and enhanced EMT characteristics.


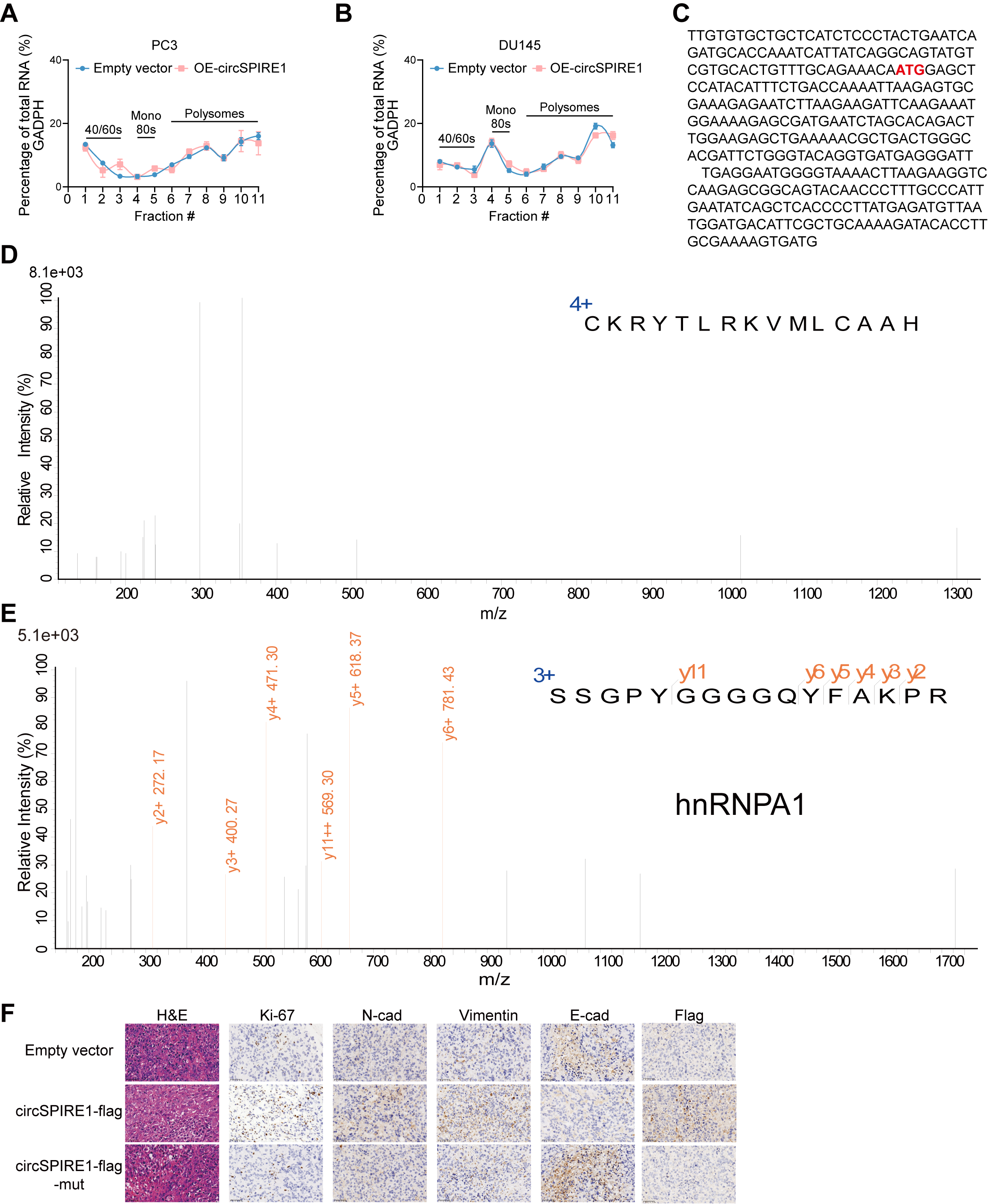


**Fig. S3 Characterization of circSPIRE1 and associated proteins**

1. B) Sucrose gradient centrifugation analysis showing the distribution of GAPDH RNA across ribosomal fractions in PC3 (A) and DU145 (B) cells. (C) Nucleotide sequence of circSPIRE1, highlighting the start codon (ATG) of the open reading frame (ORF) in red, indicating the translation initiation site. (D) Mass spectrometry analysis of the protein band excised from the silver-stained gel in Fig. 3G. The identified peptide sequence (CKRYTLRKVMLCAAH) corresponds specifically to the rtSPIRE1 protein, confirming its expression. (E) Mass spectrometry analysis of the protein band excised from the silver-stained gel in Fig. 3L. The identified peptide sequence (SSGPYGGGGQYFAKPR) corresponds specifically to hnRNPA. (F) Histological analysis of xenograft tumors in BALB/c nude mice, showing increased Ki-67, N-cadherin, and vimentin expression, along with reduced E-cadherin in circSPIRE1-flag cells compared to the Empty vector and circSPIRE1-flag-mut groups.


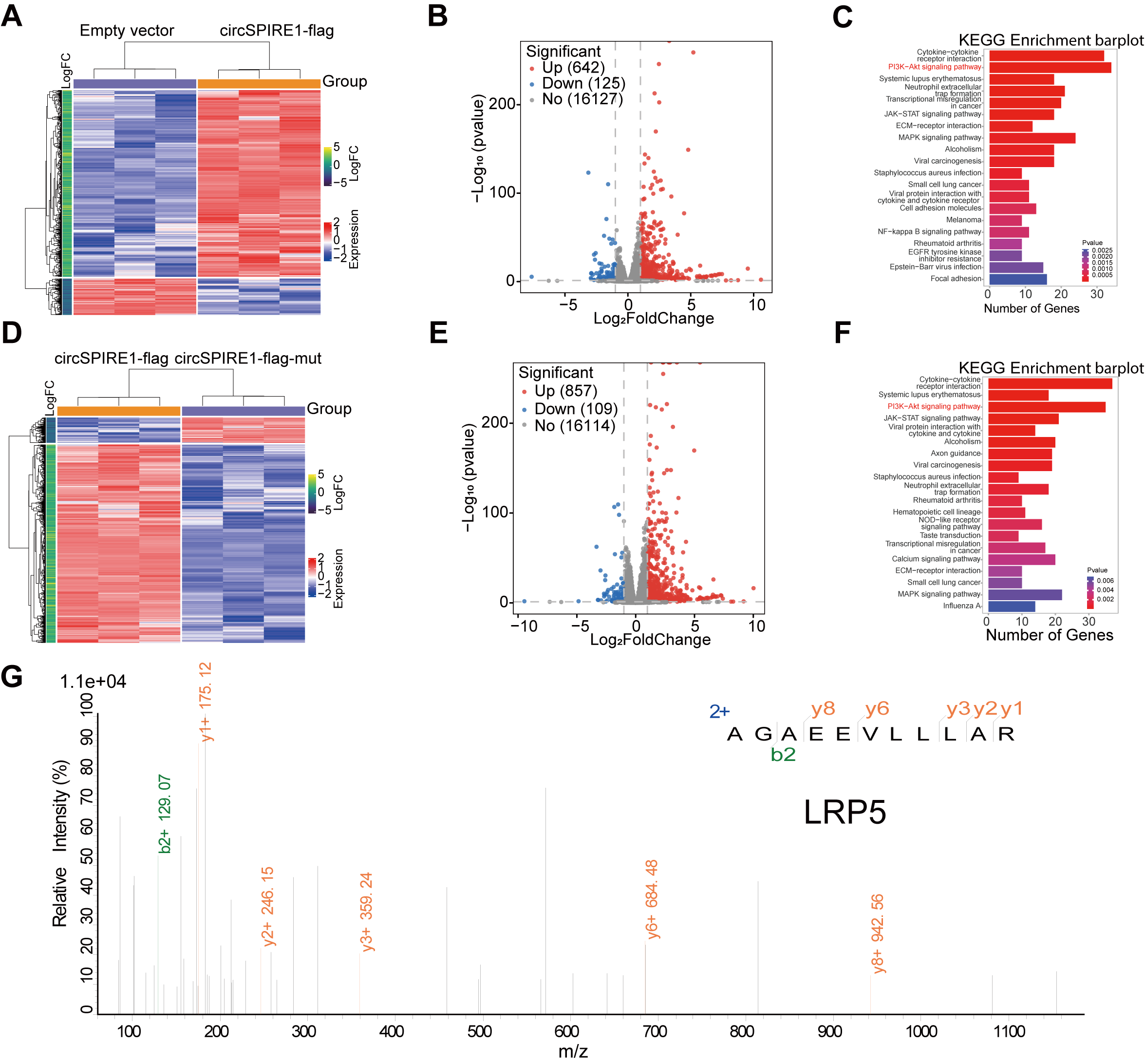


**Fig. S4 Transcriptomic and proteomic analysis of rtSPIRE1 in prostate cancer**

1. F) Heatmaps and Volcano plots from transcriptomic sequencing comparing Empty vector vs. circSPIRE1-flag and circSPIRE1-flag vs. circSPIRE1-flag-mut groups, showing differential gene expression patterns. Functional enrichment analysis identifies the PI3K/AKT signaling pathway as a top candidate activated by rtSPIRE1, n = 3 biological replicates. (G) Mass spectrometry analysis of the protein band excised from the silver-stained gel in Fig. 7H. The identified peptide sequence corresponds specifically to LRP5.


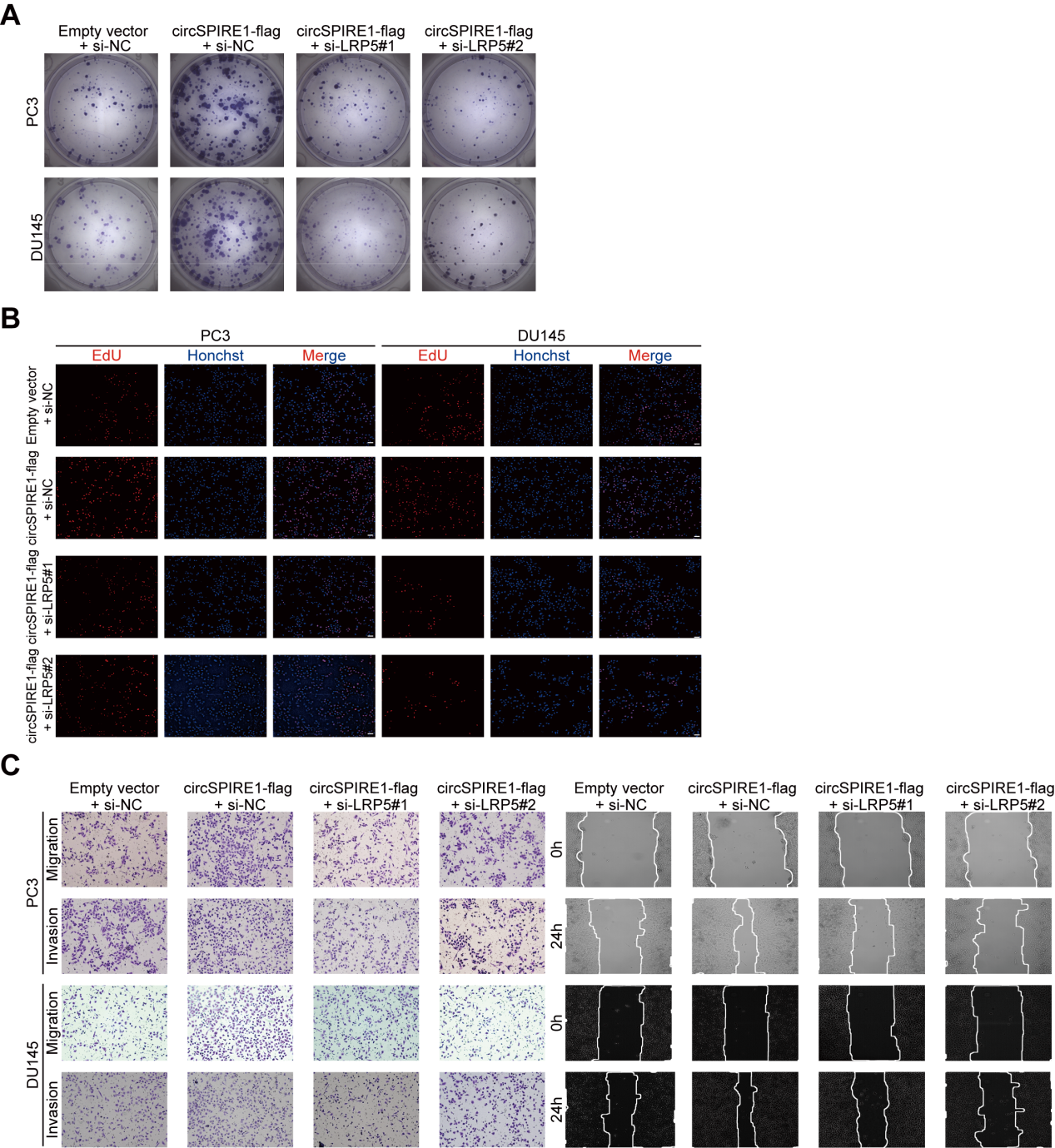


**Fig. S5 LRP5 knockdown partially reverses rtSPIRE1-driven oncogenic effects in prostate cancer**

(A) Colony formation assay showing that LRP5 knockdown partially reverses rtSPIRE1-promoted colony formation. Representative images are shown. (B) EdU incorporation assay demonstrating that LRP5 knockdown partially reverses rtSPIRE1-promoted cell proliferation. Representative images are shown. (C) Transwell migration and wound healing assays showing that LRP5 knockdown partially reverses rtSPIRE1-promoted migration and wound closure. Representative images are shown.

| **Table S1. Differential expression analysis of circRNAs in prostate cancer samples using high-throughput circRNA microarray.** | | | | | | | |
| --- | --- | --- | --- | --- | --- | --- | --- |
| ID | logFC | AveExpr | t | P.Value | adj.P.Val | B | category |
| hsa_circ_0000036 | -2.7226 | 9.9063 | -9.9852 | 0.0000 | 0.0017 | 3.7094 | down |
| hsa_circ_0000112 | -2.3703 | 7.9058 | -9.4089 | 0.0000 | 0.0020 | 3.2968 | down |
| hsa_circ_0000314 | -3.3067 | 7.7540 | -16.9503 | 0.0000 | 0.0005 | 7.2167 | down |
| hsa_circ_0000342 | -2.1702 | 7.1872 | -7.4433 | 0.0001 | 0.0040 | 1.6733 | down |
| hsa_circ_0000352 | 2.0155 | 8.8815 | 7.4310 | 0.0001 | 0.0040 | 1.6620 | up |
| hsa_circ_0000407 | -2.3982 | 9.4022 | -5.6860 | 0.0006 | 0.0084 | -0.1332 | down |
| hsa_circ_0000542 | -3.7078 | 9.1847 | -6.8407 | 0.0002 | 0.0050 | 1.0968 | down |
| hsa_circ_0000708 | -3.2076 | 9.6786 | -9.2238 | 0.0000 | 0.0021 | 3.1587 | down |
| hsa_circ_0000631 | -3.3829 | 7.5626 | -14.2595 | 0.0000 | 0.0007 | 6.1217 | down |
| hsa_circ_0000742 | -2.5554 | 10.6419 | -7.9663 | 0.0001 | 0.0033 | 2.1415 | down |
| hsa_circ_0000223 | -3.2828 | 8.3081 | -12.5689 | 0.0000 | 0.0010 | 5.2851 | down |
| hsa_circ_0000829 | 3.8247 | 10.0934 | 6.3751 | 0.0003 | 0.0060 | 0.6216 | up |
| hsa_circ_0000662 | -2.0508 | 6.9502 | -7.5108 | 0.0001 | 0.0040 | 1.7354 | down |
| hsa_circ_0001184 | -3.1287 | 7.9244 | -11.0119 | 0.0000 | 0.0013 | 4.3852 | down |
| hsa_circ_0000947 | -2.1140 | 9.1505 | -8.6145 | 0.0000 | 0.0026 | 2.6839 | down |
| hsa_circ_0001644 | -3.0931 | 9.0272 | -12.9249 | 0.0000 | 0.0010 | 5.4724 | down |
| hsa_circ_0001063 | -2.4806 | 8.0290 | -8.8748 | 0.0000 | 0.0023 | 2.8907 | down |
| hsa_circ_0001229 | 2.2429 | 9.8798 | 5.3326 | 0.0009 | 0.0099 | -0.5458 | up |
| hsa_circ_0000086 | -3.1659 | 9.1454 | -15.3763 | 0.0000 | 0.0006 | 6.6076 | down |
| hsa_circ_0001281 | 2.1177 | 7.7943 | 7.0643 | 0.0002 | 0.0047 | 1.3155 | up |
| hsa_circ_0000253 | -3.3467 | 8.2356 | -18.0282 | 0.0000 | 0.0004 | 7.5895 | down |
| hsa_circ_0000729 | -2.4943 | 7.3589 | -19.7165 | 0.0000 | 0.0004 | 8.1113 | down |
| hsa_circ_0001167 | -3.5247 | 10.0957 | -8.9313 | 0.0000 | 0.0023 | 2.9348 | down |
| hsa_circ_0000301 | -3.0156 | 10.1761 | -6.2914 | 0.0003 | 0.0062 | 0.5333 | down |
| hsa_circ_0001490 | -3.0141 | 10.9122 | -6.0210 | 0.0004 | 0.0071 | 0.2417 | down |
| hsa_circ_0001781 | -2.4392 | 8.9174 | -11.7523 | 0.0000 | 0.0011 | 4.8304 | down |
| hsa_circRNA_001838 | -2.1559 | 7.0839 | -6.2306 | 0.0004 | 0.0064 | 0.4685 | down |
| hsa_circ_0001539 | 2.2982 | 8.5899 | 6.7684 | 0.0002 | 0.0051 | 1.0247 | up |
| hsa_circ_0002368 | -3.0822 | 9.4722 | -16.6948 | 0.0000 | 0.0005 | 7.1233 | down |
| hsa_circ_0002699 | -2.0212 | 6.7427 | -5.5418 | 0.0007 | 0.0089 | -0.2994 | down |
| hsa_circ_0003090 | 2.2657 | 6.8209 | 10.1174 | 0.0000 | 0.0017 | 3.8005 | up |
| hsa_circ_0003099 | -2.1195 | 6.8905 | -9.7032 | 0.0000 | 0.0019 | 3.5107 | down |
| hsa_circ_0003408 | 2.1679 | 7.2173 | 7.0589 | 0.0002 | 0.0047 | 1.3103 | up |
| hsa_circ_0003641 | -3.9494 | 8.2221 | -8.6807 | 0.0000 | 0.0025 | 2.7370 | down |
| hsa_circ_0003907 | -3.4648 | 8.9504 | -10.9349 | 0.0000 | 0.0013 | 4.3369 | down |
| hsa_circ_0003997 | -3.5499 | 8.7340 | -11.2543 | 0.0000 | 0.0013 | 4.5346 | down |
| hsa_circ_0004077 | -3.1500 | 10.3573 | -10.0047 | 0.0000 | 0.0017 | 3.7229 | down |
| hsa_circ_0004225 | -2.9026 | 7.7824 | -16.4288 | 0.0000 | 0.0006 | 7.0239 | down |
| hsa_circ_0004300 | -2.3135 | 8.4028 | -5.9519 | 0.0005 | 0.0073 | 0.1656 | down |
| hsa_circ_0004561 | -2.8073 | 8.7338 | -8.1519 | 0.0001 | 0.0031 | 2.3009 | down |
| hsa_circ_0004796 | 2.3919 | 6.9794 | 9.7351 | 0.0000 | 0.0019 | 3.5335 | up |
| hsa_circ_0005019 | -2.4910 | 7.4981 | -7.5900 | 0.0001 | 0.0039 | 1.8076 | down |
| hsa_circ_0005048 | -2.1719 | 8.3386 | -8.2321 | 0.0001 | 0.0030 | 2.3688 | down |
| hsa_circ_0005080 | -2.7699 | 7.5706 | -5.5012 | 0.0008 | 0.0091 | -0.3467 | down |
| hsa_circ_0005187 | -3.1178 | 8.6699 | -9.3175 | 0.0000 | 0.0021 | 3.2290 | down |
| hsa_circ_0005198 | -2.8840 | 9.6140 | -6.1389 | 0.0004 | 0.0068 | 0.3700 | down |
| hsa_circ_0005255 | -2.3621 | 7.4651 | -7.8926 | 0.0001 | 0.0035 | 2.0772 | down |
| hsa_circ_0005420 | -3.4479 | 8.9152 | -9.9223 | 0.0000 | 0.0018 | 3.6656 | down |
| hsa_circ_0006169 | -2.1169 | 10.6643 | -5.5995 | 0.0007 | 0.0087 | -0.2326 | down |
| hsa_circ_0006759 | -3.1980 | 8.1976 | -19.0803 | 0.0000 | 0.0004 | 7.9229 | down |
| hsa_circ_0006773 | -2.7257 | 10.0052 | -6.7474 | 0.0002 | 0.0051 | 1.0036 | down |
| hsa_circRNA_006949 | -2.2630 | 7.8854 | -6.3076 | 0.0003 | 0.0061 | 0.5504 | down |
| hsa_circ_0007325 | -2.7583 | 8.0755 | -9.5501 | 0.0000 | 0.0020 | 3.4003 | down |
| hsa_circ_0007509 | -2.0323 | 7.2893 | -12.2715 | 0.0000 | 0.0010 | 5.1237 | down |
| hsa_circRNA_007809 | -2.4327 | 7.2253 | -6.0215 | 0.0004 | 0.0071 | 0.2423 | down |
| hsa_circ_0007877 | 2.0530 | 8.8880 | 7.1706 | 0.0001 | 0.0045 | 1.4176 | up |
| hsa_circ_0008141 | -2.0909 | 7.5063 | -7.4845 | 0.0001 | 0.0040 | 1.7113 | down |
| hsa_circ_0008168 | -2.7608 | 7.7312 | -11.5053 | 0.0000 | 0.0012 | 4.6855 | down |
| hsa_circ_0008205 | -2.7558 | 7.9644 | -12.6495 | 0.0000 | 0.0010 | 5.3281 | down |
| hsa_circ_0008219 | -2.0341 | 7.0118 | -6.0823 | 0.0004 | 0.0069 | 0.3087 | down |
| hsa_circ_0008267 | 2.1338 | 7.4362 | 7.5020 | 0.0001 | 0.0040 | 1.7273 | up |
| hsa_circ_0008410 | -2.2064 | 9.3515 | -13.8421 | 0.0000 | 0.0008 | 5.9271 | down |
| hsa_circ_0016476 | -2.8587 | 7.6746 | -11.6065 | 0.0000 | 0.0012 | 4.7453 | down |
| hsa_circRNA_018609 | -2.3380 | 7.3704 | -13.5772 | 0.0000 | 0.0009 | 5.7998 | down |
| hsa_circ_0022917 | -2.7807 | 9.6079 | -9.7728 | 0.0000 | 0.0019 | 3.5603 | down |
| hsa_circ_0022969 | -2.3930 | 7.1571 | -6.1129 | 0.0004 | 0.0068 | 0.3419 | down |
| hsa_circ_0023461 | -3.7188 | 10.1245 | -5.3612 | 0.0009 | 0.0097 | -0.5118 | down |
| hsa_circRNA_024524 | -3.1392 | 7.6196 | -9.8922 | 0.0000 | 0.0018 | 3.6445 | down |
| hsa_circRNA_025402 | 2.0822 | 7.1915 | 6.0914 | 0.0004 | 0.0069 | 0.3186 | up |
| hsa_circ_0025522 | -3.0343 | 8.9849 | -6.2212 | 0.0004 | 0.0065 | 0.4586 | down |
| hsa_circRNA_026426 | -2.8620 | 7.8601 | -6.9951 | 0.0002 | 0.0049 | 1.2484 | down |
| hsa_circ_0029301 | 2.7342 | 7.7361 | 8.9201 | 0.0000 | 0.0023 | 2.9260 | up |
| hsa_circ_0029965 | 2.5019 | 10.2491 | 5.5512 | 0.0007 | 0.0089 | -0.2884 | up |
| hsa_circ_0030509 | -2.8242 | 8.0540 | -11.1817 | 0.0000 | 0.0013 | 4.4902 | down |
| hsa_circ_0034093 | -3.1994 | 9.2861 | -12.6298 | 0.0000 | 0.0010 | 5.3176 | down |
| hsa_circ_0036906 | -2.3398 | 6.9951 | -7.0494 | 0.0002 | 0.0047 | 1.3011 | down |
| hsa_circ_0037621 | -2.7039 | 7.3853 | -24.7657 | 0.0000 | 0.0003 | 9.3207 | down |
| hsa_circ_0038011 | -3.2219 | 9.6131 | -9.7120 | 0.0000 | 0.0019 | 3.5170 | down |
| hsa_circ_0043943 | -2.6097 | 8.8336 | -8.5165 | 0.0000 | 0.0027 | 2.6044 | down |
| hsa_circ_0043944 | -3.0141 | 8.8540 | -9.1187 | 0.0000 | 0.0022 | 3.0791 | down |
| hsa_circ_0045179 | -2.2106 | 7.5221 | -12.0650 | 0.0000 | 0.0011 | 5.0088 | down |
| hsa_circ_0045799 | -2.4944 | 8.1528 | -10.5649 | 0.0000 | 0.0015 | 4.0998 | down |
| hsa_circ_0051239 | 2.6460 | 10.9247 | 8.9988 | 0.0000 | 0.0023 | 2.9871 | up |
| hsa_circ_0053944 | -3.5774 | 9.5039 | -7.4868 | 0.0001 | 0.0040 | 1.7133 | down |
| hsa_circ_0055440 | -3.6591 | 9.7040 | -9.3170 | 0.0000 | 0.0021 | 3.2286 | down |
| hsa_circ_0057041 | -2.1019 | 6.9997 | -9.7224 | 0.0000 | 0.0019 | 3.5244 | down |
| hsa_circRNA_058161 | -3.1179 | 7.6468 | -9.1095 | 0.0000 | 0.0022 | 3.0721 | down |
| hsa_circ_0060876 | -2.1571 | 8.6021 | -7.6525 | 0.0001 | 0.0038 | 1.8640 | down |
| hsa_circ_0063013 | -2.6972 | 8.5271 | -12.4291 | 0.0000 | 0.0010 | 5.2098 | down |
| hsa_circRNA_065793 | 2.0148 | 6.5382 | 8.3056 | 0.0001 | 0.0029 | 2.4304 | up |
| hsa_circ_0066596 | -3.5566 | 8.3995 | -12.2629 | 0.0000 | 0.0010 | 5.1189 | down |
| hsa_circ_0074491 | -2.1312 | 6.6197 | -11.4300 | 0.0000 | 0.0012 | 4.6406 | down |
| hsa_circ_0074559 | -2.9853 | 9.5622 | -11.8468 | 0.0000 | 0.0011 | 4.8848 | down |
| hsa_circ_0075166 | -2.4236 | 7.9352 | -10.7174 | 0.0000 | 0.0014 | 4.1986 | down |
| hsa_circ_0075549 | -2.5166 | 8.2617 | -18.0878 | 0.0000 | 0.0004 | 7.6091 | down |
| hsa_circ_0078715 | 2.0826 | 9.0765 | 6.5851 | 0.0003 | 0.0054 | 0.8392 | up |
| hsa_circ_0081481 | -3.2580 | 8.6238 | -5.5213 | 0.0008 | 0.0090 | -0.3232 | down |
| hsa_circ_0085694 | -2.3795 | 6.9992 | -13.3332 | 0.0000 | 0.0009 | 5.6796 | down |
| hsa_circ_0087800 | -2.0131 | 8.7305 | -9.6233 | 0.0000 | 0.0019 | 3.4533 | down |
| hsa_circ_0091761 | -3.2274 | 8.8984 | -8.4172 | 0.0001 | 0.0028 | 2.5230 | down |
| hsa_circ_0000368 | -3.3469 | 8.7944 | -8.0172 | 0.0001 | 0.0033 | 2.1856 | down |
| hsa_circ_0001255 | -3.6091 | 10.8665 | -7.6058 | 0.0001 | 0.0039 | 1.8219 | down |
| hsa_circ_0005039 | -2.0118 | 8.1081 | -6.1834 | 0.0004 | 0.0066 | 0.4180 | down |
| hsa_circ_0000018 | -2.1596 | 7.3985 | -9.8247 | 0.0000 | 0.0018 | 3.5970 | down |
| hsa_circ_0002720 | 2.5430 | 8.2198 | 7.2635 | 0.0001 | 0.0044 | 1.5056 | up |
| hsa_circ_0004240 | -3.6442 | 8.0238 | -11.5766 | 0.0000 | 0.0012 | 4.7277 | down |
| hsa_circ_0015278 | -2.8224 | 10.8443 | -11.6201 | 0.0000 | 0.0012 | 4.7533 | down |
| hsa_circ_0008856 | -2.4440 | 9.3330 | -8.4336 | 0.0001 | 0.0028 | 2.5365 | down |
| hsa_circ_0006956 | 2.1720 | 9.3965 | 7.0019 | 0.0002 | 0.0048 | 1.2550 | up |
| hsa_circ_0020390 | -2.3162 | 7.0094 | -5.3239 | 0.0010 | 0.0099 | -0.5562 | down |
| hsa_circ_0020926 | -2.2180 | 8.9332 | -7.5423 | 0.0001 | 0.0039 | 1.7641 | down |
| hsa_circ_0005795 | 2.5611 | 9.0156 | 6.6223 | 0.0002 | 0.0054 | 0.8772 | up |
| hsa_circ_0022378 | 2.1130 | 8.2861 | 9.3290 | 0.0000 | 0.0021 | 3.2376 | up |
| hsa_circ_0023685 | -2.8643 | 10.0302 | -13.8909 | 0.0000 | 0.0008 | 5.9502 | down |
| hsa_circ_0000374 | -2.0975 | 9.5806 | -6.0137 | 0.0005 | 0.0071 | 0.2337 | down |
| hsa_circ_0025513 | 2.1092 | 10.4865 | 5.3187 | 0.0010 | 0.0099 | -0.5624 | up |
| hsa_circRNA_101025 | -2.1480 | 8.1469 | -6.0088 | 0.0005 | 0.0071 | 0.2284 | down |
| hsa_circ_0025768 | -2.1652 | 7.9386 | -7.7672 | 0.0001 | 0.0036 | 1.9666 | down |
| hsa_circ_0009035 | 2.7037 | 7.2120 | 7.6319 | 0.0001 | 0.0038 | 1.8455 | up |
| hsa_circ_0004217 | -2.7973 | 7.8019 | -13.7214 | 0.0000 | 0.0008 | 5.8695 | down |
| hsa_circ_0005139 | -2.0768 | 9.3719 | -11.1335 | 0.0000 | 0.0013 | 4.4606 | down |
| hsa_circ_0032649 | -2.5046 | 7.5842 | -8.6530 | 0.0000 | 0.0025 | 2.7148 | down |
| hsa_circ_0005252 | -2.0995 | 7.8189 | -19.1510 | 0.0000 | 0.0004 | 7.9443 | down |
| hsa_circ_0006770 | -2.6530 | 10.5253 | -9.5407 | 0.0000 | 0.0020 | 3.3934 | down |
| hsa_circ_0034044 | -2.6964 | 11.0533 | -13.4410 | 0.0000 | 0.0009 | 5.7330 | down |
| hsa_circRNA_101464 | 2.0091 | 7.1946 | 9.9246 | 0.0000 | 0.0018 | 3.6672 | up |
| hsa_circ_0034510 | -2.3131 | 7.9482 | -15.2851 | 0.0000 | 0.0006 | 6.5696 | down |
| hsa_circ_0035277 | -3.1218 | 10.0273 | -8.1043 | 0.0001 | 0.0032 | 2.2604 | down |
| hsa_circ_0035957 | 2.0369 | 8.0734 | 7.2378 | 0.0001 | 0.0044 | 1.4813 | up |
| hsa_circ_0005745 | -2.1551 | 7.9676 | -8.1291 | 0.0001 | 0.0031 | 2.2815 | down |
| hsa_circ_0002696 | -3.1306 | 7.8654 | -16.8643 | 0.0000 | 0.0005 | 7.1855 | down |
| hsa_circ_0004018 | 2.3325 | 6.1963 | 6.7611 | 0.0002 | 0.0051 | 1.0174 | up |
| hsa_circ_0041382 | -2.1993 | 6.9986 | -15.2518 | 0.0000 | 0.0006 | 6.5558 | down |
| hsa_circ_0041555 | -2.0800 | 7.3287 | -8.6705 | 0.0000 | 0.0025 | 2.7289 | down |
| hsa_circ_0042799 | -3.0442 | 8.1824 | -5.6568 | 0.0007 | 0.0085 | -0.1666 | down |
| hsa_circ_0007990 | -2.1856 | 7.7571 | -15.1937 | 0.0000 | 0.0006 | 6.5314 | down |
| hsa_circ_0043812 | -2.1853 | 10.3746 | -5.7026 | 0.0006 | 0.0084 | -0.1142 | down |
| hsa_circ_0043947 | -3.0527 | 7.3009 | -13.1718 | 0.0000 | 0.0009 | 5.5987 | down |
| hsa_circ_0003258 | 3.1892 | 9.4720 | 10.3987 | 0.0000 | 0.0016 | 3.9903 | up |
| hsa_circ_0046854 | 2.3668 | 9.4106 | 6.3116 | 0.0003 | 0.0061 | 0.5546 | up |
| hsa_circ_0003979 | -3.2297 | 6.7061 | -21.6898 | 0.0000 | 0.0003 | 8.6394 | down |
| hsa_circ_0049271 | -2.1575 | 11.7157 | -6.8364 | 0.0002 | 0.0050 | 1.0925 | down |
| hsa_circ_0049356 | -3.3930 | 10.1190 | -12.2380 | 0.0000 | 0.0010 | 5.1052 | down |
| hsa_circ_0006446 | -2.9890 | 8.5106 | -8.8775 | 0.0000 | 0.0023 | 2.8927 | down |
| hsa_circ_0051527 | -2.3656 | 6.6197 | -15.3778 | 0.0000 | 0.0006 | 6.6082 | down |
| hsa_circ_0052318 | -3.3153 | 8.5604 | -9.6983 | 0.0000 | 0.0019 | 3.5072 | down |
| hsa_circ_0005579 | -2.0226 | 7.0606 | -9.3259 | 0.0000 | 0.0021 | 3.2353 | down |
| hsa_circ_0002938 | 2.0507 | 8.0327 | 8.0422 | 0.0001 | 0.0033 | 2.2071 | up |
| hsa_circRNA_102709 | -2.0839 | 6.8709 | -9.0195 | 0.0000 | 0.0023 | 3.0030 | down |
| hsa_circ_0055412 | -2.4866 | 7.8208 | -16.0231 | 0.0000 | 0.0006 | 6.8679 | down |
| hsa_circ_0055864 | 2.0438 | 6.8602 | 7.2844 | 0.0001 | 0.0043 | 1.5253 | up |
| hsa_circ_0056856 | -3.6341 | 9.9168 | -5.9747 | 0.0005 | 0.0072 | 0.1908 | down |
| hsa_circ_0006117 | 2.0291 | 9.3861 | 5.3531 | 0.0009 | 0.0098 | -0.5213 | up |
| hsa_circ_0060828 | -2.9496 | 8.2881 | -9.5063 | 0.0000 | 0.0020 | 3.3684 | down |
| hsa_circ_0061817 | -2.3004 | 10.3038 | -12.9427 | 0.0000 | 0.0010 | 5.4816 | down |
| hsa_circ_0001296 | -2.8289 | 11.7226 | -5.6038 | 0.0007 | 0.0087 | -0.2275 | down |
| hsa_circ_0003748 | -3.3360 | 10.1995 | -6.4526 | 0.0003 | 0.0058 | 0.7026 | down |
| hsa_circ_0008797 | -2.3420 | 10.8071 | -13.1726 | 0.0000 | 0.0009 | 5.5991 | down |
| hsa_circ_0067127 | -2.0652 | 8.4086 | -9.0720 | 0.0000 | 0.0022 | 3.0434 | down |
| hsa_circ_0068629 | 2.1060 | 7.2137 | 8.2525 | 0.0001 | 0.0030 | 2.3859 | up |
| hsa_circ_0070113 | -2.1810 | 8.7803 | -9.1752 | 0.0000 | 0.0021 | 3.1220 | down |
| hsa_circ_0071989 | -2.2546 | 10.4351 | -5.3114 | 0.0010 | 0.0100 | -0.5711 | down |
| hsa_circ_0005730 | -2.2023 | 9.0413 | -8.8938 | 0.0000 | 0.0023 | 2.9055 | down |
| hsa_circ_0001564 | -2.1098 | 7.5944 | -10.9178 | 0.0000 | 0.0013 | 4.3261 | down |
| hsa_circ_0006232 | -2.0236 | 9.0968 | -11.1395 | 0.0000 | 0.0013 | 4.4642 | down |
| hsa_circ_0076767 | -2.9460 | 10.7878 | -6.3278 | 0.0003 | 0.0061 | 0.5718 | down |
| hsa_circ_0077930 | -2.7405 | 11.8120 | -5.3622 | 0.0009 | 0.0097 | -0.5106 | down |
| hsa_circ_0079385 | -4.0923 | 10.9814 | -6.5701 | 0.0003 | 0.0055 | 0.8239 | down |
| hsa_circ_0006944 | -2.8227 | 7.6487 | -11.8663 | 0.0000 | 0.0011 | 4.8960 | down |
| hsa_circ_0005513 | -3.3017 | 8.5504 | -15.3771 | 0.0000 | 0.0006 | 6.6079 | down |
| hsa_circ_0082326 | -3.0304 | 11.4697 | -5.7742 | 0.0006 | 0.0080 | -0.0329 | down |
| hsa_circ_0001818 | -2.8227 | 10.8786 | -11.7402 | 0.0000 | 0.0011 | 4.8234 | down |
| hsa_circ_0001936 | -2.9955 | 8.5233 | -5.7592 | 0.0006 | 0.0081 | -0.0499 | down |
| hsa_circ_0002372 | -2.2292 | 6.0234 | -13.5830 | 0.0000 | 0.0009 | 5.8026 | down |
| hsa_circ_0091994 | 2.0014 | 8.0823 | 8.3834 | 0.0001 | 0.0028 | 2.4951 | up |
| hsa_circ_0001946 | -2.1460 | 9.5669 | -10.0595 | 0.0000 | 0.0017 | 3.7608 | down |
| hsa_circ_0092367 | -3.5372 | 9.2188 | -7.9865 | 0.0001 | 0.0033 | 2.1591 | down |
| hsa_circ_0092360 | -3.0672 | 10.1453 | -7.1612 | 0.0001 | 0.0045 | 1.4086 | down |
| hsa_circ_0092328 | -2.8664 | 9.6587 | -9.2809 | 0.0000 | 0.0021 | 3.2016 | down |
| hsa_circRNA_400294 | -2.2761 | 8.5405 | -6.1041 | 0.0004 | 0.0069 | 0.3324 | down |
| chr10:20534273-20534434+ | 2.0147 | 10.2970 | 8.1066 | 0.0001 | 0.0032 | 2.2623 | up |
| chr10:65326097-65359043+ | -3.0285 | 7.2903 | -22.7850 | 0.0000 | 0.0003 | 8.8999 | down |
| chr11:32610139-32611187+ | -2.4038 | 6.4973 | -12.1250 | 0.0000 | 0.0010 | 5.0424 | down |
| chr11:77051690-77060332- | -2.6615 | 8.4015 | -9.8339 | 0.0000 | 0.0018 | 3.6035 | down |
| chr12:51504659-51510203- | -2.8790 | 9.0000 | -8.6826 | 0.0000 | 0.0025 | 2.7385 | down |
| chr12:58339410-58347472+ | 2.2138 | 8.2692 | 8.3035 | 0.0001 | 0.0029 | 2.4286 | up |
| chr15:25328542-25339121+ | -2.6676 | 8.7729 | -11.1773 | 0.0000 | 0.0013 | 4.4875 | down |
| chr17:18005234-18007995+ | -2.7548 | 6.8681 | -10.2410 | 0.0000 | 0.0017 | 3.8845 | down |
| chr17:34144719-34149837+ | 2.1546 | 8.4213 | 8.2326 | 0.0001 | 0.0030 | 2.3692 | up |
| chr17:37646809-37646986+ | 2.0802 | 8.3941 | 6.6889 | 0.0002 | 0.0052 | 0.9447 | up |
| chr17:80042674-80042829- | -2.2453 | 9.3852 | -9.9872 | 0.0000 | 0.0017 | 3.7108 | down |
| chr20:18022177-18022367- | 2.4175 | 7.6854 | 8.6551 | 0.0000 | 0.0025 | 2.7165 | up |
| chr20:19560643-19566188+ | -2.8910 | 7.1952 | -12.4999 | 0.0000 | 0.0010 | 5.2481 | down |
| chr20:25477298-25481646- | 2.2781 | 7.7954 | 9.1744 | 0.0000 | 0.0021 | 3.1214 | up |
| chr22:22312863-22330179- | -2.6035 | 7.9417 | -8.9632 | 0.0000 | 0.0023 | 2.9595 | down |
| chr5:167988397-167993271- | -3.5473 | 8.1325 | -9.4404 | 0.0000 | 0.0020 | 3.3200 | down |
| chr6:21668877-21743273+ | -3.6143 | 9.6181 | -13.0624 | 0.0000 | 0.0010 | 5.5431 | down |
| chr7:91974291-92000921+ | -2.4122 | 7.4819 | -12.6400 | 0.0000 | 0.0010 | 5.3231 | down |
| hsa_circRNA_403898 | -2.3723 | 7.0806 | -6.6826 | 0.0002 | 0.0052 | 0.9383 | down |
| chr8:41466934-41469511+ | 2.0659 | 6.5226 | 10.3548 | 0.0000 | 0.0016 | 3.9610 | up |
| chr1:114248511-114254735- | -3.6842 | 8.6099 | -10.6188 | 0.0000 | 0.0015 | 4.1349 | down |
| chr1:156303463-156304503- | -2.0098 | 7.0574 | -6.2103 | 0.0004 | 0.0065 | 0.4468 | down |
| chr1:244640842-244682000+ | -2.5440 | 6.8002 | -6.7177 | 0.0002 | 0.0052 | 0.9738 | down |
| chr10:104638135-104638748+ | -2.3325 | 9.6811 | -7.8349 | 0.0001 | 0.0035 | 2.0266 | down |
| chr10:126089408-126090408- | -3.6467 | 9.4596 | -8.2028 | 0.0001 | 0.0030 | 2.3441 | down |
| chr11:3777940-3778768- | -2.8258 | 9.7393 | -11.6171 | 0.0000 | 0.0012 | 4.7515 | down |
| chr11:113933932-113935290+ | -2.5754 | 10.2276 | -7.3746 | 0.0001 | 0.0041 | 1.6097 | down |
| chr12:26147969-26156383+ | -2.1578 | 8.2140 | -7.2396 | 0.0001 | 0.0044 | 1.4831 | down |
| chr13:60485868-60548590- | -2.8590 | 7.0569 | -14.4666 | 0.0000 | 0.0007 | 6.2154 | down |
| chr13:98923104-98923600+ | -2.1656 | 7.6334 | -8.2233 | 0.0001 | 0.0030 | 2.3613 | down |
| chr14:75278441-75279301+ | -2.5496 | 8.2212 | -11.0737 | 0.0000 | 0.0013 | 4.4236 | down |
| chr16:15794592-15794782+ | -4.7556 | 9.5295 | -8.0397 | 0.0001 | 0.0033 | 2.2050 | down |
| hsa_circRNA_405535 | -2.8260 | 7.8001 | -10.2893 | 0.0000 | 0.0017 | 3.9171 | down |
| chr17:35800609-35800763+ | 3.4463 | 8.7268 | 6.0169 | 0.0005 | 0.0071 | 0.2372 | up |
| chr17:67270083-67280213- | -2.2699 | 8.9307 | -6.8805 | 0.0002 | 0.0050 | 1.1361 | down |
| chr18:44617544-44623775- | -2.6144 | 9.1867 | -7.6833 | 0.0001 | 0.0037 | 1.8918 | down |
| chr19:39328342-39329036- | -2.4066 | 8.9640 | -6.0834 | 0.0004 | 0.0069 | 0.3098 | down |
| chr19:46133214-46133304- | -2.5643 | 7.3225 | -11.5431 | 0.0000 | 0.0012 | 4.7079 | down |
| chr19:58208194-58208649- | -3.2426 | 9.4880 | -10.0959 | 0.0000 | 0.0017 | 3.7858 | down |
| chr2:32847035-32897451+ | -2.4286 | 7.3405 | -15.0339 | 0.0000 | 0.0006 | 6.4637 | down |
| chr2:122125405-122135069- | -2.9256 | 9.9172 | -10.0798 | 0.0000 | 0.0017 | 3.7747 | down |
| chr2:128934472-128935287+ | -2.7275 | 8.7968 | -7.4477 | 0.0001 | 0.0040 | 1.6774 | down |
| chr2:170671986-170677783- | -3.3357 | 7.8309 | -11.0168 | 0.0000 | 0.0013 | 4.3882 | down |
| chr2:197085692-197086934- | -2.6648 | 6.5806 | -9.8991 | 0.0000 | 0.0018 | 3.6494 | down |
| chr20:35526224-35526947- | -2.3959 | 7.7384 | -10.2361 | 0.0000 | 0.0017 | 3.8813 | down |
| chr21:45302384-45323900+ | -2.8789 | 10.4860 | -6.1111 | 0.0004 | 0.0069 | 0.3400 | down |
| chr21:47538590-47538885+ | 2.7919 | 8.1454 | 8.4797 | 0.0000 | 0.0027 | 2.5743 | up |
| chr21:47538595-47538885+ | 2.3487 | 9.8066 | 7.2683 | 0.0001 | 0.0044 | 1.5102 | up |
| chr22:28249524-28269803- | 2.8499 | 7.8726 | 8.6189 | 0.0000 | 0.0026 | 2.6874 | up |
| chr3:23299411-23301270+ | 2.3561 | 6.8938 | 5.5667 | 0.0007 | 0.0088 | -0.2705 | up |
| chr3:125170148-125176209- | -2.3889 | 6.9938 | -14.9249 | 0.0000 | 0.0006 | 6.4169 | down |
| chr3:176665094-176700237- | -3.1915 | 7.8253 | -9.2283 | 0.0000 | 0.0021 | 3.1621 | down |
| hsa_circRNA_406503 | 2.3661 | 8.0675 | 5.3151 | 0.0010 | 0.0100 | -0.5667 | up |
| chr5:647919-655699+ | -3.0108 | 8.0056 | -10.2139 | 0.0000 | 0.0017 | 3.8663 | down |
| chr6:56989531-56993638+ | -2.2155 | 7.4824 | -12.7530 | 0.0000 | 0.0010 | 5.3828 | down |
| chr7:102489106-102489847+ | -3.6078 | 8.9684 | -9.3640 | 0.0000 | 0.0021 | 3.2636 | down |
| chr9:5859467-5867902- | -2.6217 | 9.4973 | -8.7964 | 0.0000 | 0.0024 | 2.8290 | down |

| **Table S2. Clinicopathological features of prostate cancer patients used for circRNA microarray analysis (all patients underwent radical prostatectomy without prior androgen deprivation therapy).** | | | | | | |
| --- | --- | --- | --- | --- | --- | --- |
| **Patient ID** | **Age (years)** | **Sample Type** | **Gleason Score** | **TNM Stage** | **PSA (ng/mL)** | **Status** |
| SYSMH1 | 66 | Tumor / Adjacent | 7 (4+3) | T3aN0M0 | 24.6 | Alive |
| SYSMH2 | 72 | Tumor / Adjacent | 8 (4+4) | T2cN0M0 | 35.2 | Alive |
| SYSMH3 | 61 | Tumor / Adjacent | 9 (5+4) | T3bN1M0 | 58.9 | Alive |

| **Table S3. Clinicopathological features of prostate cancer patients used for qPCR validation of circSPIRE1 expression.** | | | | | | | |
| --- | --- | --- | --- | --- | --- | --- | --- |
| **Patient ID** | **Age (years)** | **Sample Type** | **Gleason Score** | **TNM Stage** | **PSA (ng/mL)** | **Status** | **circSPIRE1 Expression** |
| SYSMHP1 | 78 | Tumor / Adjacent | 7 (3+4) | T3aN0M0 | 13.2 | Alive | Upregulated |
| SYSMHP2 | 81 | Tumor / Adjacent | 8 (4+4) | T2cN0M0 | 35.6 | Dead | Upregulated |
| SYSMHP3 | 62 | Tumor / Adjacent | 9 (5+4) | T3aN0M0 | 9.5 | Alive | Upregulated |
| SYSMHP4 | 72 | Tumor / Adjacent | 10 (5+5) | T3bN1M0 | 72.3 | Alive | Upregulated |
| SYSMHP5 | 73 | Tumor / Adjacent | 8 (4+4) | T1cN0M0 | 28.7 | Alive | Downregulated |
| SYSMHP6 | 66 | Tumor / Adjacent | 7 (4+3) | T2aN0M0 | 18.9 | Alive | Unchange |
| SYSMHP7 | 64 | Tumor / Adjacent | 8(4+4) | T3bN0M0 | 62.4 | Alive | Upregulated |
| SYSMHP8 | 75 | Tumor / Adjacent | 9 (4+5) | T4N1M0 | 38.6 | Alive | Upregulated |
| SYSMHP9 | 64 | Tumor / Adjacent | 8 (4+4) | T2aN0M0 | 31.1 | Alive | Unchange |
| SYSMHP10 | 76 | Tumor / Adjacent | 7 (4+3) | T2cN0M0 | 8.7 | Dead | Upregulated |
| SYSMHP11 | 69 | Tumor / Adjacent | 9 (5+4) | T2aN0M0 | 14.5 | Alive | Upregulated |
| SYSMHP12 | 58 | Tumor / Adjacent | 7 (4+3) | T1cN0M0 | 5.3 | Alive | Unchange |
| SYSMHP13 | 74 | Tumor / Adjacent | 9 (5+4) | T2bN1M0 | 67.9 | Alive | Upregulated |
| SYSMHP14 | 63 | Tumor / Adjacent | 7 (3+4) | T3bN0M0 | 10.8 | Alive | Upregulated |
| SYSMHP15 | 69 | Tumor / Adjacent | 8 (4+4) | T3aN0M0 | 29.6 | Alive | Upregulated |
| SYSMHP16 | 61 | Tumor / Adjacent | 8 (4+4) | T2cN0M0 | 7.1 | Alive | Upregulated |
| SYSMHP17 | 70 | Tumor / Adjacent | 7 (4+3) | T2aN0M0 | 19.4 | Alive | Downregulated |
| SYSMHP18 | 67 | Tumor / Adjacent | 9 (5+4) | T3bN1M0 | 81.2 | Alive | Upregulated |
| SYSMHP19 | 69 | Tumor / Adjacent | 9 (5+4) | T2bN0M0 | 11.6 | Alive | Upregulated |
| SYSMHP20 | 72 | Tumor / Adjacent | 8 (4+4) | T3aN0M1 | 33.3 | Alive | Upregulated |

| **Table S4. The relationship of circSPRE1 expression levels (ΔCt) in prostate cancer tissues with clinicopathological parameters (n=80).** | | | | | | |
| --- | --- | --- | --- | --- | --- | --- |
|  |  |  |  |  |  |  |
| **Variables** |  | **Number (%)** |  | **Mean ± SD** |  | ***P*-value** |
| **Age (year)** |  |  |  |  |  |  |
| < 65 |  | 27 (33.8%) |  | 5.76±2.02 |  | 0.394 |
| ≥ 65 |  | 53 (66.2%) |  | 5.47±2.28 |  |  |
| **T stage** |  |  |  |  |  |  |
| T1 ~ T2 |  | 46 (57.5%) |  | 5.11±2.31 |  | 0.029 |
| T3 ~ T4 |  | 34 (42.5%) |  | 6.11±1.73 |  |  |
| **N stage** |  |  |  |  |  |  |
| N 0 |  | 60 (75.0%) |  | 5.56±2.17 |  | 0.851 |
| N 1 |  | 20 (25.0%) |  | 5.46±2.04 |  |  |
| **M stage** |  |  |  |  |  |  |
| M 0 |  | 58 (72.5%) |  | 5.30±2.08 |  | <0.001 |
| M 1 |  | 22 (27.5%) |  | 7.51±1.54 |  |  |
| **Gleason score** |  |  |  |  |  |  |
| ≤ 7 |  | 39 (48.8%) |  | 4.14±1.59 |  | <0.001 |
| > 7 |  | 41 (51.2%) |  | 6.87±1.68 |  |  |
| **Stage** |  |  |  |  |  |  |
| I |  | 20 (25.0%) |  | 4.18±1.69 |  | <0.001 |
| II |  | 25 (31.2%) |  | 4.80±2.25 |  |  |
| III |  | 17 (21.2%) |  | 5.72±1.66 |  |  |
| IV |  | 18 (22.5%) |  | 7.07±1.50 |  |  |

| **Table S5. The relationship of rtSPIRE1 expression levels (ΔCt) in prostate cancer tissues with clinicopathological parameters (n=100).** | | | | | | |
| --- | --- | --- | --- | --- | --- | --- |
|  |  |  |  |  |  |  |
| **Variables** |  | **Number (%)** |  | **Mean ± SD** |  | ***P*-value** |
| **Age (year)** |  |  |  |  |  |  |
| < 65 |  | 35 (35.0%) |  | 15.60±7.21 |  | 0.358 |
| ≥ 65 |  | 65 (65.0%) |  | 17.05±7.92 |  |  |
| **T stage** |  |  |  |  |  |  |
| T1 ~ T2 |  | 36 (36.0%) |  | 10.72±4.13 |  | <0.001 |
| T3 ~ T4 |  | 64 (64.0%) |  | 19.81±7.27 |  |  |
| **N stage** |  |  |  |  |  |  |
| N 0 |  | 72 (72.0%) |  | 16.12±7.43 |  | 0.412 |
| N 1 |  | 28 (28.0%) |  | 17.61±8.32 |  |  |
| **M stage** |  |  |  |  |  |  |
| M 0 |  | 77 (77.0%) |  | 15.76±7.53 |  | 0.074 |
| M 1 |  | 23 (23.0%) |  | 19.13±7.77 |  |  |
| **Gleason score** |  |  |  |  |  |  |
| Gleason 6 |  | 11 (11.0%) |  | 7.54±1.74 |  | <0.001 |
| Gleason 3+4 |  | 20 (20.0%) |  | 11.74±2.79 |  |  |
| Gleason 4+3 |  | 35 (35.0%) |  | 14.77±3.41 |  |  |
| Gleason ≥ 8 |  | 35 (35.0%) |  | 24.01±7.41 |  |  |

| **Table S6. List of primer sequences used in the study.** | | | |
| --- | --- | --- | --- |
| **Name** |  |  | **Sequence (5’ to 3’)** |
| **For quantitative real-time PCR** | | | |
| human GAPDH |  | forward | TGCACCACCAACTGCTTAGC |
|  |  | reverse | GGCATGGACTGTGGTCATGAG |
| circSPIRE1 |  | forward | TCGCTGCAAAAGATACACCT |
|  |  | reverse | GAGATGAGCAGCACACAACAT |
| hsa_circ_0005039 |  | forward | GGAGAGGGCCTGTAGGGCAT |
|  |  | reverse | ACGGCTAGGCCTCCGTGA |
| hsa_circ_0003099 |  | forward | GAGTGTGTCAGTGATGATGATT |
|  |  | reverse | TGGAGCCTGAGAAGACAAGG |
| hsa_circ_0016476 |  | forward | ATTTTTAAGTGAGAAGGGT |
|  |  | reverse | GGCTTTTGGTTTCTTCCTGC |
| hsa_circ_0025402 |  | forward | CACTTATTAGCTCAGCCACAG |
|  |  | reverse | CTAGAGGCACATTCCGACCC |
| hsa_circ_0038011 |  | forward | TTTGATGACCGCAGCAGGTGGA |
|  |  | reverse | CTGGGACGGCTTTAAACACC |
| hsa_circ_0082326 |  | forward | GTGTGGCTGGGCGTGTAGATAA |
|  |  | reverse | GCAACATCCCAAATCGGTCT |
| SPIRE1 |  | forward | TGAAACCAACTCCACCACGG |
|  |  | reverse | GTGGCCGCATTGCTAATCTG |
| U6 |  | forward | TGCTCGCTTCGGCAGCACAT |
|  |  | reverse | CTTGCGCAGGGGCCATGCTA |
| hY1 |  | forward | TGGAAGCCAGTGCCTGTCATGT |
|  |  | reverse | AGCCGACTCGAAGCTGGAGGT |
| CDR1as |  | forward | CCCAGTCTTCCATCAACTGGCT |
|  |  | reverse | AAGACCTTGACACAGGTGCCAT |
| circANRIL |  | forward | GCTGGGATTACAGGTGTGAGACACC |
|  |  | reverse | GAATCAGAATGAGGCTTATTCTTCTCATC |
| LRP5 |  | forward | GGACACCAACATGATCGAGTCG |
|  |  | reverse | CGCTCAATGCTGTGCAGATTCC |
| **FISH probe** | | | |
| circSPIRE1-FISH |  | Cy3-GGGAGATGAGCAGCACACAACATCACTTTTCGCAAGGTGT | |
| **RNA pulldown probe** | | | |
| circSPIRE1-RNA pulldown |  | GATGAGCAGCACACAACA+TCACTTT+TCGCAA | |
| NC-RNA pulldown |  | TTGCGAAAAG+TGATGT+TGTGTGC+TGCTCATC | |
| **siRNA** | | | |
| siNC |  | UUCUCCGAACGUGUCACG | |
| circSPIRE1-siRNA#1 |  | UUGCGAAAAGUGAUGUUGUTT | |
| circSPIRE1-siRNA#2 |  | GUGAUGUUGUGUGCUGCUCTT | |
| si-hnRNPA1 |  | AATGGGGAACGCTCACGGACT | |
| si-Renilla |  | UAUAAGAACCAUUACCAGAUUUGCCUG | |
| LRP5-siRNA#1 |  | GGACCGACCUGGACACCAATT | |
| LRP5-siRNA#2 |  | GGAUCUCCCUCGAGACCAATT | |
